# Evolutionary analysis of transcription elongation factors reveals conserved and lineage-specific regulatory domains

**DOI:** 10.1371/journal.pbio.3003855

**Published:** 2026-06-15

**Authors:** Alex M. Francette, Aakash Grover, Nathan Clark, Karen M. Arndt

**Affiliations:** 1 Department of Biological Sciences, University of Pittsburgh, Pittsburgh, Pennsylvania, United States of America; 2 Department of Cell Biology and Physiology, Washington University School of Medicine, St. Louis, Missouri, United States of America; National Cancer Institute, UNITED STATES OF AMERICA

## Abstract

In eukaryotes, transcription elongation factors (TEFs) associate with RNA Polymerase II (RNAPII) to facilitate gene expression and couple transcription to co-transcriptional processes, including chromatin regulation and RNA processing. To further our understanding of TEF biology, we developed a domain-centric analysis pipeline to perform a broad survey of 10 TEF orthologs—Paf1, Ctr9, Cdc73, Rtf1, Leo1, Spt4, Spt5, Spt6, Spn1, and Elf1—across the Tree of Life and analyze their evolutionary patterns in a structural context. We report evidence for all 10 TEFs being present in the last eukaryotic common ancestor, indicating that mechanisms of TEF-mediated transcription regulation are both ancient and conserved. However, some early-diverging eukaryotic clades exhibit signs of altered TEF domain composition. A comparative phylogenetic analysis highlighted conserved regions of TEFs that are detected in both metazoans and fungi and other regions that appear clade-specific, detected only in metazoans. These observations, together with additional insights generated from evolutionary rate covariation analysis, shed light on under-characterized aspects of TEFs, including domains for which functions have yet to be dissected.

## Abstract 2

Transcription elongation factors associate with RNA Polymerase II to regulate gene expression and coordinate chromatin regulation and RNA processing. This study presents an evolutionary analysis of ten core transcription elongation factors and shows that all ten factors originated in the last eukaryotic common ancestor and display both conserved and lineage-specific domains.

## Introduction

In all three domains of life—Bacteria, Archaea, and Eukaryota—genomic information is expressed in the form of RNA through the process of transcription. In eukaryotes, protein-coding genes are transcribed by the multi-subunit RNA Polymerase II (RNAPII) and its associated factors. Transcription elongation factors (TEFs) are one class of accessory factors that engage with active RNAPII to coordinate co-transcriptional events and promote RNAPII elongation through chromatin. Insights into TEF-mediated activity have been primarily derived from studies performed in eukaryotic model systems, such as fungi (e.g., *Saccharomyces cerevisiae*, *Schizosaccharomyces pombe*), invertebrates (e.g., *Drosophila melanogaster*, *Caenorhabditis elegans*), vertebrates (e.g., *Mus musculus*), and angiosperms (e.g., *Arabidopsis thaliana)* [1–4]. From decades of study, several critical TEFs have been identified. Core TEFs, defined as those shared amongst major model organisms and visualized by cryo-EM of the activated transcription elongation complex called EC* include: Spt4(hSUPT4H) and Spt5(hSUPT5H) comprising DSIF, Spt6(hSUPT6H), Spn1(hIWS1), Elf1(hELOF1), and the five-subunit Polymerase Associated Factor 1 Complex or Paf1C—Paf1(hPAF1/PD2), Ctr9(hCTR9), Cdc73(hCDC73/Parafibromin), Rtf1(hRTF1), and Leo1(hLEO1) [4]. For simplicity, these orthologs will be referred to by their *S. cerevisiae* protein designations.

The discovery and characterization of functionally conserved, often modular TEF domains have uncovered roles in promoting appropriate transcription. TEFs impact RNAPII elongation rate, are required for processive transcription in vivo, facilitate the transient removal and re-deposition of histones after RNAPII passage, and direct post-translational modifications to RNAPII and histones [1–6]. However, the extent to which TEF sequences are conserved across diverse species remains unclear. In this study, we investigated the presence, organization, and conservation of these factors in diverse organisms to gain insights into the mechanisms of transcription regulation across the Tree of Life.

Modern analyses classify eukaryotes into several major lineages, many of which harbor divergent or poorly characterized transcriptional machinery. These lineages include the supergroups of Amorphea (e.g., Amoebozoa, Animalia, Fungi), Archaeplastida (Viridiplantae, Red Algae, Glaucophytes), Cryptista (e.g., *Cryptomonas*), CRuMs (Collodictyonid, Rigifilida, and *Mantamonas*), Discoba (e.g., *Trypanosoma, Euglena*), Haptista (e.g., *Choanocystis*), Metamonada (e.g., *Giardia, Trichomonas*), and TSAR (Telonemids, Stramenopiles, Alveolates, and Rhizaria; *e.g.,* brown algae, *Plasmodium*, *Toxoplasma*) [7]. The loosely defined “Excavate” groups of Discoba, Metamonada, and Malawimonadae and the orphan group of Ancyromonada are considered some of the more basal lineages of eukaryotes, placing them in an important position to understand the acquisition or loss of TEFs throughout evolution [7–9].

A limited number of TEFs are shared between prokaryotes and eukaryotes, and several studies have characterized TEF homologs in single-celled eukaryotes [10]. Spt5 (NusG in bacteria) is the only TEF previously identified in all domains of life [11,12]. In Archaea and Eukaryota, Spt5 dimerizes with a small, globular protein, Spt4 [13], and Elf1 homologs have also been detected within both clades [10,14]. Spt6 shares structural homology with the bacterial transcription accessory factor Tex [11,15–17]. Recently, Paf1 and Spt6 have been characterized as deeply conserved in eukaryotes [18]. However, the conservation of Spn1 and the remaining Paf1C subunits is less understood. Several investigations into eukaryotic TEFs outside of animals, fungi, and plants have been performed. Studies in *Trypanosoma brucei* found evidence for orthologs of Spt4, Spt5, Spt6, Ctr9, Cdc73, and Leo1 [19]. Another study reported Elf1 orthologs in Apicomplexa species including the obligate intracellular parasite, *Toxoplasma gondii*, which are proposed to be functionally divergent [20]. There are some indications that specific TEFs are not ubiquitous throughout Eukaryota, particularly in some parasites. A BLASTP search across 11 distantly related eukaryotes, including members of TSAR, Discoba, Metamonada, and Amoebozoa, failed to identify Paf1C subunits in Metamonads (*Trichomonas* and *Giardia*) [19]. However, from the remaining species, Cdc73 was detected in all, and Ctr9 was found in all but *Plasmodium falciparum*. Paf1, Leo1, and Rtf1 were variably identified in this search. In another study, precipitation of RNAPII-associated factors in *Trypanosoma brucei* failed to identify Paf1, Rtf1, Spn1, or Elf1 [21]. More recently, BLASTP and TBLASTN searches of Microsporidia species (an intracellular parasitic sister clade to fungi) variably identified homologs of Spt5, Spt6, and Paf1C [22]. In essence, the landscape of elongation factor conservation is unclear, and it remains unknown whether the Last Eukaryotic Common Ancestor (LECA) had some or all these factors.

Seeking a deeper, unified analysis of their conservation and structural heterogeneity, we investigated the presence and co-occurrence of 10 core TEFs in the proteomes of 304 broadly diverged species. Our analysis of TEF domain architecture suggests that the composition and general form of these TEFs were mostly defined in the LECA. However, we also identify multiple instances of apparent divergence in the composition of transcription elongation machinery. Furthermore, we compared the conservation of residues between orthologous proteins in fungal and metazoan proteomes to identify specific residues and regions of common and clade-specific importance. These findings home in on recently identified protein-protein interaction interfaces and identify additional regions of potential functional importance. Together, our analysis provides both broad and in-depth insights into the conservation and function of factors fundamental to eukaryotic transcription.

## Results

### Paf1C and Spn1 are only detected in eukaryotes

**Fig 1:**
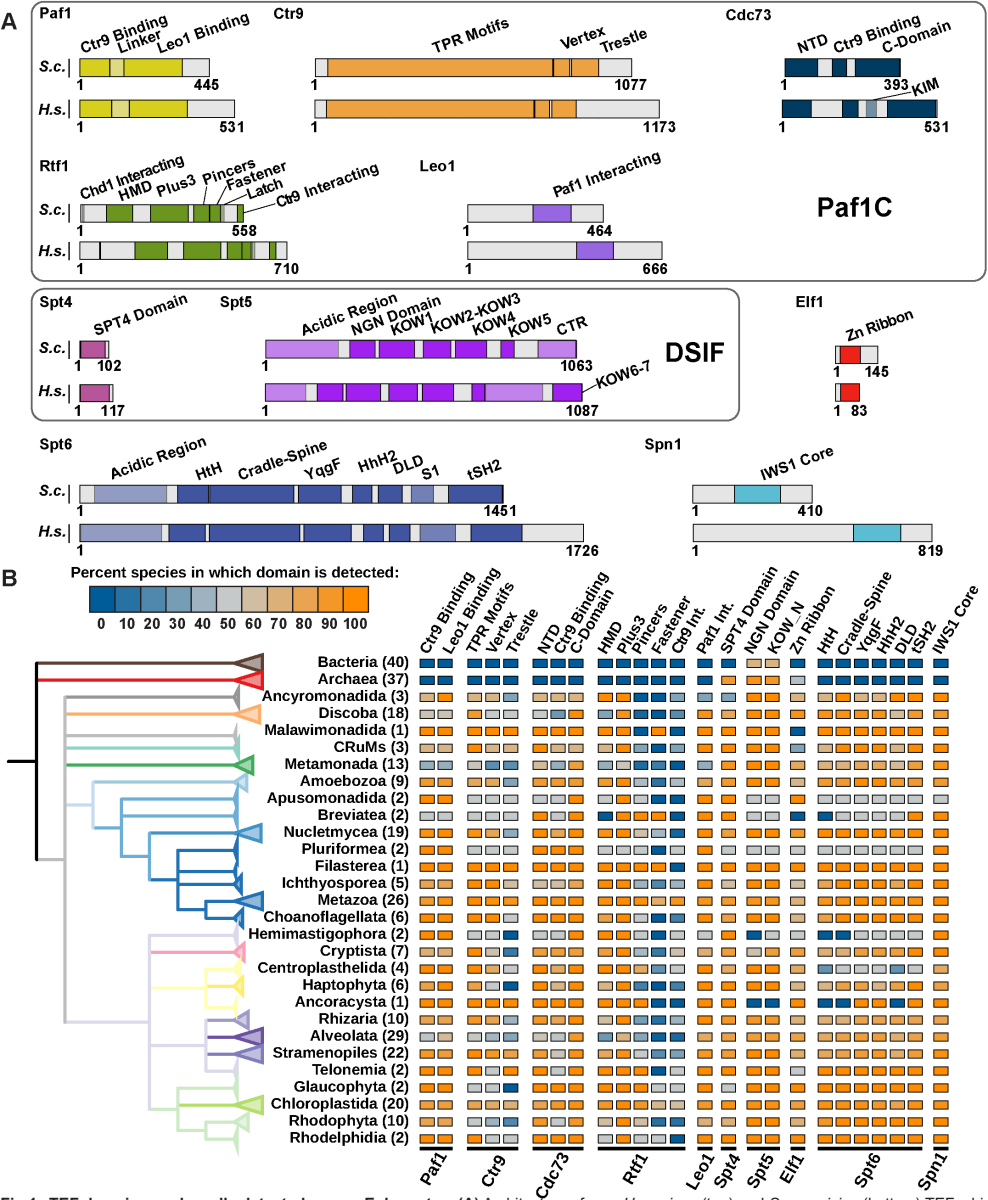
TEF domains are broadly detected across Eukaryotes. **(A)** Architecture of core *H. sapiens* (top) and *S. cerevisiae* (bottom) TEFs, highlighting regions with verified functions. Domains utilized for homolog searches are in dark shading. Additional domains too small or unstructured to be confidently used in homolog searches are highlighted with light shading. **(B)** Distribution of TEF domain detection across the Tree of Life. The number of species analyzed in each clade is shown in parentheses. Branch points reflect taxonomic classifications provided by GTDB and EukProt. Each column represents the percentage of detection across clades for the indicated domain via HMM scan. Unless otherwise mentioned, the Fastener indicated in Rtf1 corresponds to the RNAPII-fastener as defined in [25]. Location of data files in the Zenodo repository used to generate plots in panel 1B has been provided in S2 Table. TPR - Tetratricopeptide Repeat; NTD, N-Terminal Domain; HMD, Histone Modification Domain; NGN, NusG N-terminal; KOW, Kyrpides-Ouzounis-Woese; CTR, C-Terminal Repeat; HtH, Helix-Turn-Helix; HhH2, double-helix-hairpin-helix; DLD, Death-Like Domain; tSH2, Tandem Src Homology 2.

Since several TEFs are structurally modular and contain more than one identifiable domain (Fig 1A), some orthologous proteins may not share the exact structural organization of known TEFs. Previous investigations searching for TEF orthologs along the entire protein sequence may have been biased against those with divergent architectures [11,19]. Therefore, to minimize this bias, we took a domain-centered approach to identify orthologs of Paf1, Ctr9, Cdc73, Rtf1, Leo1, Spt4, Spt5, Spt6, Spn1, and Elf1 across a diversity of bacterial (*n* = 37), archaeal (*n* = 40), and eukaryotic (*n* = 227) proteomes (Figs 1A and S1) (Materials and methods). Although hWDR61/ySki8, a WD40-domain protein in the SKI complex that interacts with the RNA exosome, has been identified in both *H. sapiens* and *A. thaliana* Paf1C [23,24], we have excluded this factor from our analyses. Its similarity to other non-redundant WD40-domain proteins and the lack of an additional diagnostic domain make it difficult to confidently detect WDR61 in highly diverged species.

In our study, a “domain” is operationally defined as a functional unit of a protein supported by structural, genetic, and/or biochemical evidence, irrespective of its ability to form a stable fold. This definition limits the utility of existing resources such as structure-based Encyclopedia of Domains [26] and the PFAM database [27] in which some defined protein domains span multiple regions with distinct functions. For example, the Paf1 family (PF03985) defined in PFAM includes regions interfacing with Ctr9, RNAPII, and Leo1 [27]. Thus, we adopted a customized strategy to capture a wider range of putative orthologs with defined domain boundaries and without the *a priori* assumption that the structural organization (i.e., architecture) of TEFs is invariant.

We searched proteomes from the EukProt and GTDB databases for each protein of interest using both BLAST, with whole protein sequences as templates, and custom hidden Markov models (HMMs) built with each domain (see Materials and methods). The resulting hits were then individually inspected and filtered to remove spurious identifications, a process guided by a combined analysis of multiple sequence alignments (MSAs), gene tree reconstructions, and further searches using both PFAM-provided and custom HMMs. This methodology, informed by extensive structural and functional studies of the target proteins, was designed to be a sensitive survey that captures orthologs that may have diverged significantly over time. All domain-specific searches outperformed BLAST, allowing expansion of the range of identified orthologs (S2 and S3 Figs). We note that our ability to detect a domain using this pipeline in any given proteome is limited by several factors including: (1) the source of the predicted proteome either from transcriptomic profiling or genomic sequence (S4A Fig); (2) the completeness of the predicted proteome (S4B and S4C Fig); (3) the amount of information in the HMM for the domain (S4D Fig); and (4) the sequence divergence of the target domain in a given proteome.

Using this domain-centric pipeline, we recapitulate observations made in previous studies focused on the evolution of transcription factors [11,28]. Spt5, identified by the presence of the NGN domain and at least one KOW domain, is the only TEF universally conserved across kingdoms (Figs 1B and S5). Spt4 orthologs, identified by their zinc-finger (hereafter, the SPT4 Domain; Fig 1A), are detected in most archaea and eukaryotes. Elf1 orthologs, characterized by the presence of an Elf1 zinc-finger (Fig 1A), are detected in most of the Asgardarchaeota, some non-Asgard archaeal species, and most eukaryotes.

Spt6 domains, Paf1C domains, and the Spn1 IWS1 domain were only detected in eukaryotes. Given the structural similarity between the Spt6 and Tex core domains, we expected that our search would identify Tex homologs from prokaryotic proteomes in addition to Spt6 homologs from eukaryotes. However, using our HMMs, we were only able to detect Spt6 homologs in eukaryotes (Figs 1B and S5). These observations indicate that while extant Tex and the core of Spt6 are derived from a common ancestor, over the course of evolution, the core domains in Spt6 have diverged from Tex at the sequence level. Our inability to detect Paf1C domains or the Spn1 IWS1 domain in any prokaryotic lineage prompted us to ask if we could detect these domains in a larger set of 218 Asgardarchaeota proteomes (S4C Fig), the closest known archaeal clade to eukaryotic species [29]. While we were able to detect Spt5, Spt4, and Elf1 orthologs (S4E Fig), we were unable to detect any proteins that contained the IWS1 domain or Paf1C domains, even in this targeted search. Thus, we propose that Paf1C and Spn1 are eukaryotic innovations.

### Some Paf1C domains are not detected in sub-clades of Discoba, Metamonada, and Alveolata

**Fig 2:**
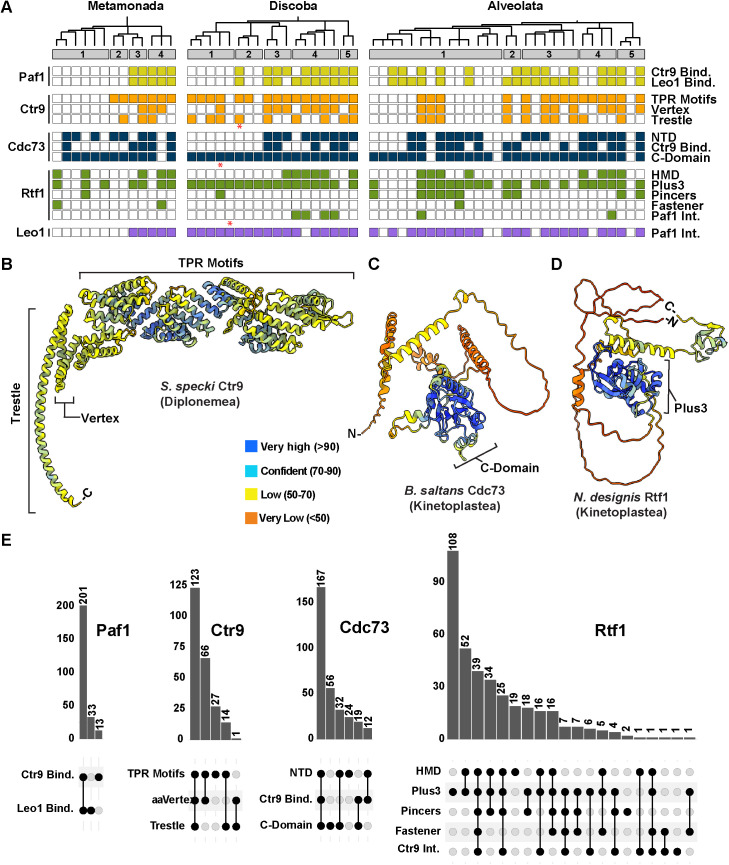
Paf1C domains are variably detected in discobids, metamonads, and alveolates. **(A)** Binary presence-absence heatmaps for Paf1C domain detection in Discoba, Metamonada, and Alveolata. Dendrogram on top represents phylogenetic relationship between species, as shown in S5 Fig. Filled boxes indicate that the domain was detected by proteome HMM search for the examined species. Gray bars represent sub-clades within the three clades. Metamonda: (1) BF clade (Fornicata and Barthelona), (2) Parabasalia, (3) Anaeramoebidae, and (4) Preaxostyla. Discoba: (1) Kinetoplastea, (2) Diplonemea, (3) Euglenida, (4) Heterolobosea, (5) Jakobida. Alveolata: (1) Apicomplexa, (2) Colpodellida, (3) DP clade (Dinoflagellata and Perkinsea), (4) Ciliophora, (5) Colponemidae. **(B–D)** AlphaFold3 predicted structures of Paf1C orthologs from indicated clades (marked by red asterisks in panel (A) colored by pLDDT score. **(B)** Model of *S. specki* (Diplonemea) Ctr9, indicating the presence of the Vertex in this clade. **(C)** Model of *B. saltans* (Kinetoplastea) Cdc73. The NTD is not detectable in proteomes from this clade. **(D)** Model of *N. designis* (Kinetoplastea) Rtf1. The HMD is not detected in proteomes from this clade. **(E)** UpSet plots depicting the coincidence of domain detection in homologs of indicated proteins as determined by HMMER hmmscan [30] using custom HMMs. Scan domain eValue (--domE) threshold set to 10^−3^. Location of data files in the Zenodo repository used to generate plots in this figure has been provided in S2 Table.

Most TEF domains were detected in basal eukaryotes, suggesting that these TEFs were present in the LECA (S5 Fig). However, while some TEFs, like Spt4, Spt5, Spt6, and Spn1, were almost universally detected across eukaryotic clades, the detection of some domains in Paf1C and the Elf1 domain was less consistent, reflecting possible domain-loss events or highly divergent sequence composition (S5 Fig). The under-detection of the Elf1 domain did not display any clade-specific pattern (S5 and S6A Figs). In contrast, several Paf1C domains were not detected in specific discobid, metamonad, and alveolate sub-clades (2A and S6A Figs). We observed two patterns of domain under-detection. First, in some sub-clades, we were unable to detect any domain corresponding to the proteins of interest (Fig 2A). For example, we were unable to detect the Ctr9-binding and the Leo1-binding domains of Paf1 in Kinetoplastea and Parabasalia, sub-clades of Discoba (gray box 1) and Metamonada (gray box 2), respectively. Second, in some sub-clades, we detected at least one but not all domains corresponding to the protein (Fig 2A). For example, in the Kinetoplastea and Diplonemea sub-clades of Discoba (gray boxes 1 and 2, respectively), we detected proteins containing the Rtf1 Plus3 domain but not the Rtf1 HMD.

Our inability to detect some domains in these clades can be attributed to extreme sequence divergence, a legitimate evolutionary loss event, the overall lower completeness of predicted proteomes (S6B Fig), low numbers of representative species for certain subclades, or a technical limitation of our approach. We therefore used alternative strategies to search for Paf1C domains in clades in which our HMM search was unable to find a given domain (S1 Table). Alternative searches were carried out in a hierarchical manner. First, FoldSeek was used to search the AlphaFold-predicted structure database of UniProt proteomes [31–33]. If FoldSeek did not detect the domain of interest in a given clade, we predicted the AlphaFold3-structures of sequences from that clade that were identified through any HMM search using our original pipeline. These structures were manually inspected for the missing domain. In cases where this approach did not detect the domain, we performed PSI–BLAST searches of NCBI proteomes in the clades of interest. Lastly, in cases where none of the three alternative searches were successful in detecting a domain, we used our domain-specific HMMs to probe additional proteomes corresponding to the clade of interest obtained from the NCBI database (see Materials and methods).

In our HMM-based search, the Rtf1 HMD and Cdc73 NTD were not detected in the Colpodellida sub-clade of alveolates (Fig 2A, gray box 2). In contrast, FoldSeek was able to detect these domains within this sub-clade (S1 Table). Similarly, the Ctr9 Vertex was not identified in the Parabasalia and Diplonemea sub-clades by our search. However, AlphaFold3 structure predictions of Ctr9 orthologs identified by our HMM and belonging to this sub-clade indicate that this domain is indeed present (Fig 2B and S1 Table). The Leo1-binding domain in Paf1 was not detected in the Fornicata sub-clade (5 proteomes) of metamonads by our HMM-based search of the EukProt database (Fig 2A and S1 Table), FoldSeek, AlphaFold3, and PSI–BLAST searches. However, using HMM-based searches of Fornicata proteomes available in the NCBI database (*n* = 14), we were able to detect this domain in one fornicate species (S1 Table).

Manual inspection of AlphaFold3 predicted structures of Cdc73 homologs in Kinetoplastea and Diplonemea indicate that while the Cdc73 C-Domain was detected in these sub-clades, an NTD was not detected with these methods (example shown in Fig 2C). Similarly, the Rtf1 HMD was not evident in predicted structures of Rtf1 homologs in these two clades, although the Plus3 domain was readily detectable (example shown in Fig 2D). These observations are consistent with our HMM search, which was unable to detect the Rtf1 HMD and Cdc73 NTD in Kinetoplastea and Diplonemea sub-clades (S1 Table and Fig 2A). These approaches were also unable to detect the Paf1 Leo1-binding domain in the Parabasalia sub-clade of Metamonada (gray box 2) and the Kinetoplastea sub-clade of Discoba (gray box 1). Altogether, these results indicate that select sub-clades of unicellular eukaryotes may have lost some Paf1C domains or the sequences of these domains are too diverged to be detected by the employed methods.

### Multi-domain architectures of TEFs are broadly conserved

A striking feature of TEFs like Rtf1, Spt5, Paf1, Cdc73, Ctr9, and Spt6 is their modular, multi-domain architecture. To determine if domain architecture is conserved for these six TEFs, we collected the identified homologs for each TEF and asked which of the domains are detected in each homolog by HMM scans. We then represented these data as UpSet plots (Figs 2E, S7A, and S7B). We find that, in general, for all multi-domain TEFs except Rtf1, the most commonly detected domain architecture is one in which all known domains are detected. In the case of Rtf1 (Fig 2E), the most common domain architecture is the one in which only the Plus3 domain is detected. The next most common architecture for identified Rtf1 homologs is the co-occurrence of the HMD and Plus3 domain. The lack of detection of other Rtf1 domains (Pincers, Fastener, and the Ctr9-interacting region) in the identified homologs might relate to the fact that the HMMs of these domains have relatively lower information content than the two other Rtf1 domains (S4D Fig).

### Conservation score analysis pinpoints functionally conserved residues in TEFs

**Fig 3:**
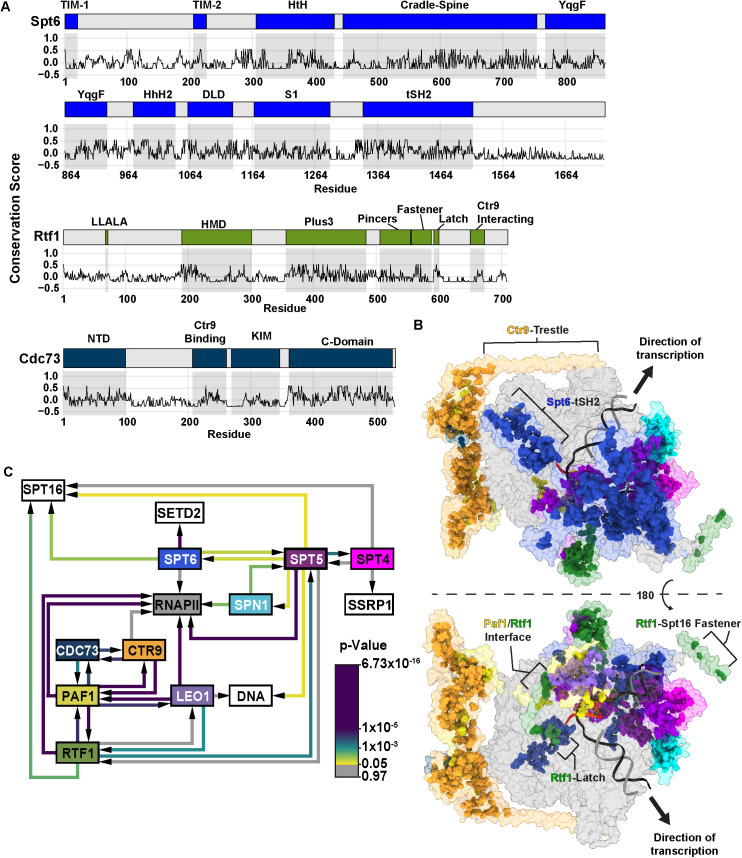
Conserved regions in TEFs. **(A)** Conservation scores were calculated from relative evolutionary rates (RERs) as follows: (−log10(RER + 0.1)) of Spt6, Rtf1, and Cdc73 across each residue along the *H. sapiens* homolog. Residues with conservation scores > 0 are more conserved than average, and those < 0 are less conserved than average across a given protein (see Materials and methods). The domain map of each protein is stacked on top of a line plot showing the conservation score of each residue. Gray boxes in the line plot highlight domain boundaries. **(B)** Structure of transcription elongation complex (PDB: 9EH2) with atoms of conserved residues (conservation score > 0.28) shown as spheres. Nucleic acids are represented as ribbons and RNAPII surface is shown in gray. SPT16, SSRP1, and SETD2 are hidden for clarity. The structure was prepared in the absence of human Elf1 homolog, ELOF1. **(C)** Network of pairwise interactions between TEFs and other components of the transcription elongation complex. The average conservation scores of surface-exposed residues within the interfaces (TEF residues within 4 Å of the interacting molecule and solvent-accessible surface area >50 Å^2^) were compared to all surface-exposed residues in the protein by Signed Rank Test to determine if residues at a given interface have a conservation score significantly higher than those of other non-buried residues. Arrows originate from a TEF towards its interactor. Edge colors indicate p-value. Since conservation scores were not calculated for any RNAPII subunit, SPT16, SSRP1, or SETD2, no edges emerge from these nodes. Location of data files in the Zenodo repository used to generate plots in this figure has been provided in S2 Table.

A slow rate of amino acid substitution, i.e., a high degree of conservation, in orthologous proteins can suggest the functional importance of specific residues. Using our dataset of TEF orthologs, we calculated conservation scores for residues along human TEF sequences (see Materials and methods). As expected, previously characterized functional regions are generally more conserved than other regions, though not uniformly so (Figs 3A and S8A).

Mapping conserved residues onto a recent structure of the human transcription elongation complex (PDB: 9EH2, lacking Elf1) [34] identifies the locations of inter- and intra-protein contacts (Figs 3B and S8B). A conservation score threshold >0.28 was chosen to selectively highlight regions of known functional importance. For example, core residues stabilizing structured domains, such as the Spt6 tSH2 domain (Fig 3B), are markedly more conserved [35]. Regions with defined regulatory roles are also highly conserved, such as the Rtf1 Latch, which stimulates RNAPII elongation rate (Figs 3A, 3B, and S8A) [25]. In contrast, structurally conserved regions that lack strongly conserved residues likely serve primarily architectural roles. For example, few residues in the Ctr9 trestle exhibit particularly high conservation (Figs 3B and S8A). Despite this observation, the Ctr9 trestle is detected by the HMM scan in 60% (138/231) of CTR9 homologs spread broadly across the Tree of Life (Figs 2E and S5).

To systematically describe how macromolecular interfaces dictate evolutionary patterns in TEFs, we examined every binary interaction resolved in the 9EH2 structure (Figs 3C and S8C) [34]. We assessed whether surface-exposed residues within 4 Å of other proteins or DNA in this static view exhibit a higher-than-average conservation score relative to other surface-exposed residues of each TEF. We find that Paf1 is not only a central hub for interfaces with other Paf1C subunits, but that these interfaces are all significantly enriched for conserved residues from both sides of the interaction (Fig 3C). For example, our analysis indicates specific residues at the interface between the Rtf1 Fastener and Paf1 Linker are more conserved relative to other structurally resolved residues in these domains (Fig 3B and 3C). Likewise, the residues of Paf1, Rtf1, Leo1, and Spt5 proximal to RNAPII are highly conserved (Fig 3C).

Importantly, less conserved residues do not imply a lack of functional importance when there is positive selection or relaxed selective constraints such as the case with intrinsically disordered charge blocks in the Ctr9 C-terminal tail [36]. Spt4, a small, globular protein with a deeply conserved interaction with Spt5 [13], provides another example. Given the compact nature of Spt4, much of its sequence is comprised of nearly invariant residues like the cysteine residues coordinating Zn^2+^ in its Zn-finger motif (S8A and S8B Fig) [37]. Since conservation score is normalized over the length of each protein, the conservation of these residues may drive down the relative conservation of residues at the Spt4/Spt5 interface. In essence, when most residues are extremely conserved, few seem more conserved than average. Together, this analysis indicates that the interfaces between Paf1C subunits and several points of attachment to RNAPII are conserved across species.

### Conserved residues in fungi and metazoans suggest regions with preserved functions

While *S. cerevisiae* is a prominent model for the study of eukaryotic transcription, the common ancestors of fungi and metazoans diverged approximately 1 billion years ago [38]. Therefore, an important objective is to understand which TEF features are shared between fungi and humans and which now differ. We reasoned that if we compared the independent patterns of evolution of TEF orthologs in metazoan- and fungal-specific proteome databases, we could identify residues and structural elements that are concordantly conserved across both clades. Alternatively, we expected to identify residues and structural features that are more conserved in either clade. These regions would likely represent functional elements unique to or selectively lost from one clade.

**Fig 4:**
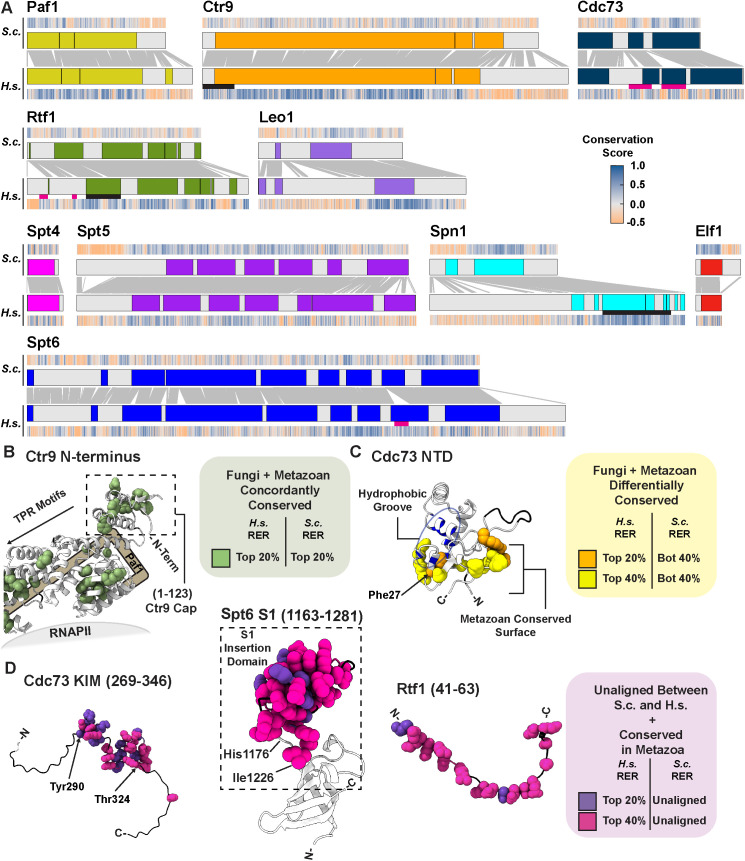
Similarities and divergences between metazoan and fungal TEF homologs highlight broad and clade-specific sites of potential functional importance. **(A)** Pairwise residue alignment (gray lines) of indicated proteins mapped on the linear domain maps of *S. cerevisiae* (top) and *H. sapiens* (bottom) homologs. Domains are as indicated in Figs 3A and S8A. Heatmaps represent the clade-specific conservation score of each residue for the indicated homolog. For *S. cerevisiae* and *H. sapiens*, conservation score was calculated using the alignment and gene trees built from fungal and metazoan homologs, respectively. Lines below the domain maps denote regions that are highlighted in panels B–D and S9B–S9D Fig, with black lines indicating conserved regions in both fungi and metazoan homologs and pink lines indicating conserved regions in metazoans that are not mappable in the fungal homologs. **(B–D)** AlphaFold2 predicted structures of human TEFs, highlighting different classes of conserved residues. Residues unaligned between the *S. cerevisiae* and *H. sapiens* orthologs and are not well conserved have been colored black. **(B)** Concordantly conserved residues (top 20% conserved residues in metazoan and fungal homologs) in the N-terminus of Ctr9. **(C)** Differentially conserved residues (top 20%–40% conserved residues in metazoan homologs and bottom 40% conserved residues in fungal homologs) in Cdc73 NTD. Phe27 and residues labeled in blue form a hydrophobic surface groove that was previously suggested to be a potential binding surface [40]. Residues in Cdc73 NTD selectively conserved in metazoans include Lys15, Lys16, Phe27, Asp109, Arg110, Ser111, Ala 112, Pro113, and Glu115. **(D)** Top 20%–40% conserved residues in metazoan homologs that could not be aligned to the *S. cerevisiae* homolog. Conserved residues are highlighted in Cdc73 (left), an insertion within the S1 domain of Spt6 (middle), and near the Rtf1 N-terminus (right). Location of data files in the Zenodo repository used to generate plots in this figure has been provided in S2 Table.

To this end, we examined RefSeq metazoan and fungal proteome databases [39] for TEF orthologs. While our detection of TEFs in basal eukaryotes is variable (Fig 1B), the divergence of fungi, such as *S. cerevisiae,* from metazoans, including *H. sapiens,* occurred comparatively recently leading to a high frequency of detection for all factors using a standard BLAST search (S9A Fig). Next, we calculated the conservation scores for fungal and metazoan orthologs and mapped *S. cerevisiae* TEF residues to *H. sapiens* sequences (Fig 4A). We then examined patterns of TEF evolution in the context of the *H. sapiens* AlphaFold structural predictions (Figs 4B–4D, S9B–S9D, and S10).

We first identified residues occupying homologous positions in the primary structures of TEFs that are amongst the most conserved in both fungi and metazoans (top 20%) (Figs 4B and S9B). These residues are expected to confer an evolutionary disadvantage when mutated in either *H. sapiens* or *S. cerevisiae* and thus likely mediate a shared role in Opisthokonts, which include both fungi and metazoans. As anticipated, we find core residues deep within protein folds, such as the IWS1 core domain in Spn1, in this class (S9B Fig, left). However, this conservation in Spn1 extends C-terminally from the structured core into a space proximal to the sites where Elf1 and the Spt5 NGN bind to elongating RNAPII. This stretch was recently shown by cryo-EM to be directly interacting with human Elf1, Spt5 NGN, and the Rpb2 subunit of RNAPII [5,6]. We also identify residues extending from the Rtf1 HMD region that fasten to the histone chaperone Spt16 [34,41] to be highly conserved in both clades (S9B Fig, right).

The Ctr9 TPR motifs scaffold Paf1C assembly, mediating extensive interactions with Paf1 and Cdc73 [42–44]. Interestingly, the N-terminal cap of Ctr9, consisting of two β-strands, a short α-helix, and the first TPR motif, is enriched with residues highly conserved in both fungi and metazoans (Fig 4B). While yeast Ctr9 contains a 16 residue N-terminal tail that interacts with Paf1 and is important for Paf1-Ctr9 subcomplex assembly [42], there is no such extension in metazoan Ctr9. This cluster of conserved, solvent-facing residues in the Ctr9 cap is consistent with an additional role as a potential platform for interactors outside of Paf1C. Together, this analysis provides a global view of the conserved architectural cores and interaction surfaces maintained across evolutionary timescales.

### Differential conservation score analysis uncovers putative interfaces selectively conserved in Metazoa but not in Fungi

To explore how TEF biology may differ between fungi and metazoans, we investigated the possibility that structurally homologous regions in TEFs may be repurposed between clades. For example, if a purely architectural sequence acquires a new function as the site of an intermolecular interface, we would expect the identity of the residues involved in that specific interaction to change less over time. Therefore, we searched for residues that are slow to change in metazoans while rapidly changing in fungi. We categorized residues as differentially conserved if they are amongst the highest 20% or 40% conserved in *H. sapiens* and the lowest 40% conserved in *S. cerevisiae* (Figs 4C and S9C).

This analysis identified a highly localized cluster of residues that are variable in fungi but remain strongly conserved in human Cdc73 (Fig 4C). These residues are almost entirely distinct from a previously reported hydrophobic groove in the NTD speculated to be an interaction interface [40]. Similarly, we note that a region of the Paf1 linker, between its known interacting sites with Rtf1 and Leo1, contains several differentially conserved residues, suggesting an uncharacterized role in metazoans (S9C Fig, left). We additionally observe that the solvent-exposed surface of Ctr9 TPR motifs harbors two sites with differentially conserved residues, one of which lies in close proximity to the binding site of WDR61 (S9C Fig, right) [23]. The other site contains residues within TPR repeats 2–8 and lies just downstream of the N-terminal cap. These data highlight currently under-characterized regions in TEFs that might drive metazoan-specific functions or potentially mediate ancestral functions that have been lost in fungi.

### Sites of high conservation within regions unaligned between *H. sapiens* and *S. cerevisiae* TEFs

To survey core TEFs for potential functional features not observed in *S. cerevisiae*, we examined *H. sapiens* TEF sequences that failed to align to their *S. cerevisiae* counterparts for regions of high conservation (conservation score in highest 40%). With respect to Paf1C, we find that the Cyclin-K Interacting Motif (KIM) of Cdc73 (residues Tyr290-Thr324), recently shown to directly interact with CDK12/13 [45], is selectively identified out of the larger disordered Cdc73 linker to be highly conserved (Fig 4D, left). As another example, the linker sequence between the NTD and Ctr9-binding domains in Cdc73 contains clusters of conserved residues in metazoans (S9D Fig, left). Although some of these regions have defined functions as nuclear/nucleolar localization signals (residues 125–139 and 192–194, respectively) [46,47], the region between Arg147 and Glu180 is additionally enriched for conserved residues (S9D Fig, left). These residues overlap with Cdc73 residues 128–227, a site previously found to facilitate the association of Cdc73 with the H3 K9 methyltransferase, SUV39H1 [48]. We also find two sites in human Rtf1 stand out as hotspots of conservation in areas that do not align to *S. cerevisiae* (Fig 4D, right and S9D Fig, right). One spans residues 41–63 immediately preceding the LLALA box (Fig 4D), a site required for interaction between Rtf1 and homologs of the chromatin remodeler Chd1 [49]. The other site (residues 149–162) lies between the Rtf1 LLALA box and the HMD (S9D Fig, right). We predict these regions to contribute towards important functions of Paf1C in metazoans.

Intriguingly, whereas yeast and most other clades have a contiguous S1 domain fold in Spt6, the human protein exhibits a bipartite architecture bearing a 50-residue insertion, which we term the S1 Insertion Domain (SID) (Fig 4D, middle). This region was recently resolved in a cryo-EM structure of a human transcription elongation complex [34] and had previously been noted in a comparison of S1-domain containing proteins as a peculiarity in the *Caenorhabditis elegans* Spt6 ortholog, EMB-5, which is absent in other S1 proteins [50]. The SID is predicted to adopt a C2HC zinc-finger fold (S11A Fig) and is juxtaposed to the RNA-exit channel in the RNAPII elongation complex [25,34]. However, the SID has an electronegative surface likely incompatible with RNA binding (S11B Fig). Searches for similar inserts across eukaryotic S1 domains revealed the SID region to be ubiquitous in metazoans and variably detected in choanoflagellates (S11C and S11D Fig), a sister group to Metazoa [51]. Other eukaryotic species show no such insertion, excepting outliers amongst Alveolata and Metamonada (S11C and S11D Fig). Thus, we conclude that the SID is not a universal feature of Spt6 but potentially originated prior to the divergence between choanoflagellates and metazoans.

### Support for a functional Ctr9-interacting motif in human Rtf1

Unlike in available metazoan elongation complex structures, a C-terminal region of fungal Rtf1, which we term the Hook, can be resolved to associate with Ctr9 as a key attachment point to Paf1C [52–54]. Crosslinking evidence places residues of the human Rtf1 Hook near Ctr9 on transcribing RNAPII [25], however, metazoan Rtf1 is biochemically dissociable from Paf1C while fungal Rtf1 is a more stable member of Paf1C [55–58]. Intriguingly, near the C-terminus of human Rtf1, we find a stretch of conserved residues (655–674) that failed to align to the *S. cerevisiae* Rtf1 Hook region but aligned to the presumed Hook of other fungal Rtf1 orthologs (Figs 4A, S12A, and S12B). These residues in human Rtf1 have been previously reported as homologous to the fungal Ctr9-interacting region [53]. Indeed, deleting the C-terminal residues from human Rtf1 containing this region (604–710) leads to reduced immunoprecipitation of other Paf1C subunits [59]. These observations led us to examine the possibility that the human Rtf1 Hook interacts with Ctr9 using the same binding modality as yeast.

AlphaFold3 predictions suggest both human and yeast Rtf1 consistently fold onto the same groove of Ctr9 (S12C–S12E Fig), albeit with the human pair having a slightly lower confidence. Furthermore, Ctr9 from both *S. cerevisiae* and *H.* sapiens bears a lipophilic pocket that appears biochemically compatible with the lipophilic face of the Rtf1 Hook (S12F and S12G Fig). These data support a model where Rtf1 in metazoans, including humans*,* utilizes a linear motif to bind Ctr9.

### Evolutionary rate co-variation landscape of TEFs

**Fig 5:**
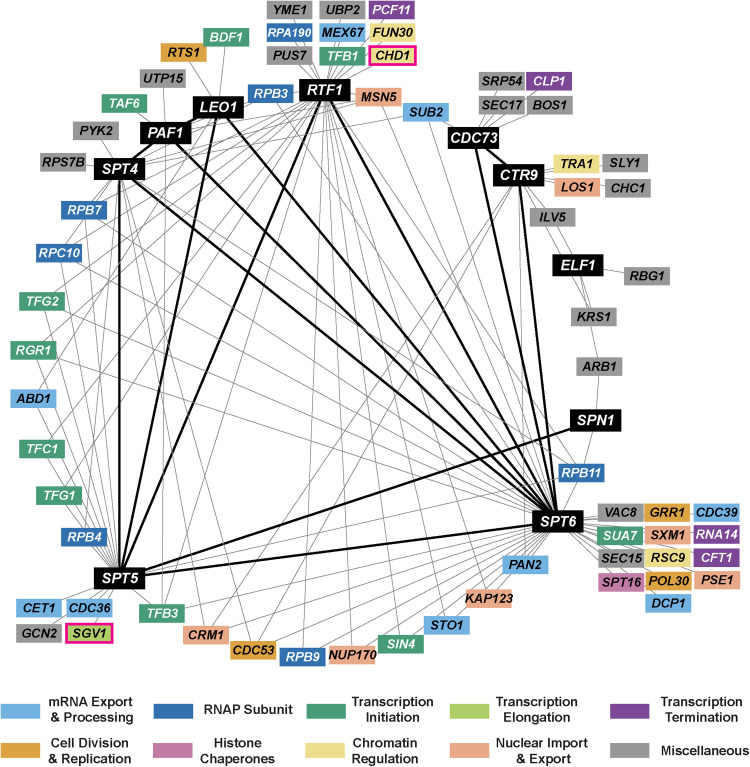
ERC analysis identifies known and putative functional interactors of TEFs. Each node in the network represents a gene, and an edge between two nodes indicates that the ERC between the two genes has a *Z*-score greater than 3.5. Black nodes represent TEFs, and black edges represent connections among TEFs. ERC values between genes in this network range from 7.6 to 16.0. Color of nodes represents the function attributed to the gene. Miscellaneous functional categories include metabolism (*PYK2, ILV5, PUS7*), protein homeostasis (*YME1, UBP2*), protein trafficking (*VAC8, CHC1, SRP54*), ribosome subunits and biogenesis (*RPS7B, ARB1*), rRNA processing (*UTP15*), translation (*KRS1, RBG1, GCN2*) and vesicle trafficking (*SEC15, SLY1, SEC17, BOS1*). Boxed in pink are nodes of interest that are discussed in the main text. Location of data files in the Zenodo repository used to generate plots in this figure has been provided in S2 Table.

Our evolutionary analysis of TEFs has identified several domains and residues that are candidates for functional characterization in future studies. However, previous work indicates that co-variation of whole-protein evolutionary rates between genes (evolutionary rate covariation; ERC) is another powerful predictor of functional interaction amongst proteins undergoing shared selective pressures [60,61]. Although the ability of genes to undergo multiple simultaneous selective pressures means that not every known physical and functional interaction is represented as high ERC pairs, genes that do share high ERC have successfully been validated to functionally interact in a variety of contexts [62–66]. We therefore utilized a dataset generated from 343 yeast species to identify genes whose whole-protein evolutionary rates co-vary with TEFs. As done previously, we standardized the ERC values for each TEF by calculating *Z*-scores [67]. For this analysis, we considered an arbitrary *Z*-score cut-off of 3.5 to identify genes that have a “high” ERC with TEFs. We then visualized genes that have a high ERC with the 10 candidate TEFs as a network (Fig 5). Consistent with their known physical and functional association, TEFs shared a high ERC with each other (black nodes connected with black edges). The interconnectedness of nodes in this network indicates that several genes share a high ERC with the same TEFs, resulting in a clustering coefficient much higher than expected from randomly generated networks of similar size (S13A Fig). These genes include those that encode core RNAPII subunits and transcription initiation factors.

As predicted, several known functional interactors of TEFs share a high ERC with them. Here, we highlight two examples (nodes highlighted by pink outlines in Fig 5). First, the chromatin remodeler *CHD1* has a high ERC with *RTF1* (*Z*-score_*RTF1-CHD1*_ = 3.88). As noted above, the LLALA box in Rtf1 directly interacts with the CHCT domain of Chd1 and is required for proper localization of Chd1 on gene bodies [49,68,69]. Second, *SGV1*/*BUR1* has a high ERC with *SPT5* (*Z*-score_*SPT5-SGV1*_ = 3.56). Bur1 phosphorylates the C-terminal repeat region of Spt5, and this phosphorylation is required for the proper recruitment of Paf1C onto gene bodies [70–73].

Using the *Z*-score cut-off, we also note that compared to other TEFs, a greater number of genes have a high ERC with *SPT6* and *RTF1* (S13B Fig)*.* Additionally, a high proportion of genes co-varying with *SPT6, RTF1,* and *CDC73* are unique to these TEFs (S13B Fig), suggestive of individualized functions not generally shared between TEFs. These include, but are not restricted to, genes encoding several transcription termination factors (Pcf11, Clp1, Rna14, and Cft1; purple nodes in Fig 5). Overall, this analysis highlights both known and underappreciated functions of TEFs and identifies putative functional interactors that warrant further experimental characterization.

## Discussion

### TEFs are broadly conserved across eukaryotes with notable exceptions

**Fig 6:**
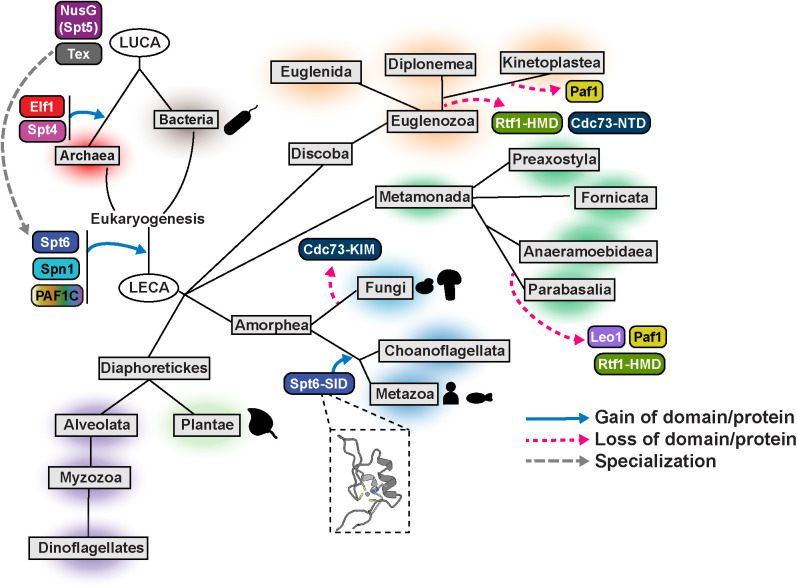
Model of TEF domain evolution. Proposed model reflecting the acquisition and putative loss of TEF domains across clades in the Tree of Life. Spt5 is the only known universal elongation factor. Spt4 and Elf1 are archaeal innovations. Spt6, Spn1, and Paf1C are eukaryotic elongation factors. Though broadly conserved, we were unable to detect specific Paf1C domains in some eukaryotic clades. Spt6 homologs in choanoflagellates and metazoans have gained a zinc finger domain we termed the SID.

This study provides a comprehensive analysis of core TEF orthologs in 304 species distributed across the Tree of Life to understand variations and conserved features of eukaryotic transcription regulation (Fig 6). Our findings demonstrate the deep evolutionary origins of TEFs, reveal key architectural and regulatory features that have been maintained for over a billion years, and uncover lineage-specific innovations. As expected, we find Spt5 in all domains of life and Spt4 and Elf1 orthologs in both Archaea and Eukaryota. All subunits of Paf1C and Spn1 appear to be eukaryote-specific. Their presence in early branching eukaryotic lineages indicates establishment prior to the LECA. Although the hWDR61/ySki8 subunit of Paf1C was not examined in this work, the inclusion of WDR61 in both human and plant Paf1C [23,24] argues that this interaction is ancient, preceding the divergence of Opisthokonta and Viridiplantae. ySki8 has not been identified as part of *S. cerevisiae* Paf1C, which would be consistent with the loss of this interaction in at least some fungi.

Paf1C subunits were less consistently detected by HMM search, particularly in discobid, metamonad, and alveolate proteomes. The reasons for the apparent dispensability of Paf1C domains within some clades remain unclear. However, previous analyses set precedent that even the most deeply conserved TEF, NusG, has been lost in some bacterial endosymbionts [74]. In the case of the Rtf1 HMD, this variability may be linked to the key role of the HMD in stimulating H2B monoubiquitylation (H2Bub) [69,75,76]. We speculate that altered chromatin biology and transcriptional regulation in these clades may relax the evolutionary constraints coordinating H2Bub with transcription elongation, leading to the divergence or loss of the Rtf1 HMD. Furthermore, in clades where the HMD was not detected, alternative mechanisms may have evolved to compensate for the putative loss of HMD-dependent functions. For example, in Opisthokonts, H2Bub is required for co-transcriptional H3K79 methylation by the Dot1 methyltransferase [77–79]. In kinetoplastids, a sub-clade of Discoba in which the HMD is putatively lost (Fig 2A and S1 Table), Dot1 orthologs have been reported to operate independently of H2Bub [80].

### A repertoire of conserved TEF features

Mapping conservation scores onto protein structures yields functional maps of TEFs that suggest testable mechanisms. While we demonstrate that this analysis can sensitively highlight known protein-protein interfaces (Fig 3B and 3C), we find no such conservation hotspots along the length of the Ctr9 trestle. These observations suggest this helix is primarily an architectural feature or acts to sterically occlude portions of RNAPII from other factors, though further work is needed to test these functions. Comparative conservation score analyses additionally identify concordantly and discordantly conserved regions of fungal and metazoan TEFs. We also identify several poorly understood structural features of TEFs, including regions along Paf1, Ctr9, Cdc73, Rtf1, and Spt6 (Fig 4). As a resource for future studies, ChimeraX structural visualization session files highlighting conserved regions in these proteins have been provided (see Data availability statement) [81]. This evolutionary framework should accelerate hypothesis-driven mutagenesis experiments, unify findings across model systems, and guide the discovery of lineage-specific mechanisms of transcription elongation regulation.

### The under-characterized features of the transcription elongation complex

TEFs have been studied for decades, yet our results indicate that there remains much to explore. Amongst the most conspicuous and uncharacterized regions in metazoan TEFs are the Spt6 SID, Cdc73 NTD, and the N-terminal extension from the Rtf1 LLALA box. Combining our insights with analyses enabled by powerful methodologies such as targeted AlphaFold multimer screens [82], site-specific crosslinking [83], and classical biochemical and genetic approaches will be crucial to clarify the specific biological functions of TEFs.

ERC analyses of yeast species further suggest underexplored connections in the evolutionary patterns shared amongst TEFs (Fig 5). Intriguingly, evolutionary rates of *SPT6*, *RTF1*, and *SPT5* co-vary with a high number of genes, and many genes exhibit evolutionary rates uniquely co-varying with *RTF1*, *SPT6*, or *CDC73* (S13B Fig). We propose two non-exclusive models to explain our observations. First, these TEFs might serve as multifunctional hubs in the elongation complex, bridging the physical or functional interactions of diverse proteins with the transcription elongation machinery. This is consistent with several studies implicating the requirement of Spt6, Rtf1, and Cdc73 for proper transcription termination [2,84–87]. Second, these TEFs might play an important role in other cellular processes not directly related to transcription elongation. In support of this model, *SPT6* has a high ERC with *POL30* (*Z*-score_*SPT6-POL30*_ = 3.66), which encodes the DNA polymerase processivity factor PCNA [88]. Consistent with this observation, Spt6 is required for DNA replication and genome stability maintenance [2,86,89,90]. We speculate that high ERC pairs such as *RTF1* and *FUN30,* which encodes a chromatin remodeler, or *LEO1* and *RTS1,* which encodes a regulatory subunit of PP2A phosphatase, represent candidate functional interactors with the elongation machinery.

While informative, our approach is limited in its ability to parse function from rapidly evolving disordered regions, such as those involved in histone interactions or phase separation [36,91,92]. Additionally, several reported interactions, including those between Spt6 and Spn1 [93–95] and between Cdc73 and Spt6 [96,97], are unresolved in the 9EH2 PDB structure and as such remain unexamined in this study. Nevertheless, this work provides a rich, evolution-guided roadmap for future mechanistic studies to understand conserved and divergent principles of transcription regulation.

## Materials and methods

### Data retrieval and domain annotations

Domain annotations were manually curated from published crystal or cryo-EM structures of *H. sapiens* or *S. cerevisiae* proteins or, when these were not available, from structures predicted by AlphaFold. Predicted protein structures that were precomputed using AlphaFold2 and subjected to energy minimization were downloaded from the AlphaFold Protein Structure Database (December 12, 2023; RRID:SCR_023662) [31,32]. Domain boundaries and the sources of their annotations are provided in Zenodo repository (see Data availability statement; RRID:SCR_004129).

Prokaryotic proteome sequences were retrieved from the Genome Taxonomy Database (GTDB) (release 214, April 21, 2023) [98–101]. For the combined EukProt/GTDB proteome search, Archaeal (*n* = 37) and Bacterial (*n* = 40) proteome sequences representing a range of prokaryotic clades were manually subset from GTDB to be included in the search. Only Prokaryotic proteomes annotated as “GTDB species representatives” were considered. Eukaryotic proteomes (*n* = 227) were retrieved from the EukProt Database (Version 3, November 22, 2022) [102]. Eukaryotic species selected included the full “Comparative Set”, which had been curated to represent maximum breadth and proteome completeness [102].

For a deeper search within Asgardarchaeota, 218 proteome sequences were subset from the same GTDB release. Additional RefSeq annotated Fungal (*n* = 128) and Metazoan (*n* = 706) proteomes (assembly level—chromosome and complete) were downloaded from NCBI RefSeq (January 9, 2025) [103]. Species lists of each proteome set are provided in Zenodo repository (see Data availability statement).

### Domain-forward search for elongation factor homologs

Searches for orthologs of human TEFs were performed using an initial permissive BLAST+ (v2.13.0; RRID:SCR_004870) [104] against proteomes from GTDB and EukProt (-evalue 10). The hits with the lowest *e*-value for each species were then aligned using MUSCLE (v5.1; RRID:SCR_011812) [105] (-super5). Next, we individually searched for the domains of each protein through the proteome database. To do this, we first trimmed the relevant MSA to the boundaries of the domain of interest as annotated in the *S. cerevisiae* and *H. sapiens* orthologs of interest using a custom script. Entries from species that showed no protein sequence over the portion of the MSA containing that domain were purged from the domain-trimmed MSA using seqkit (v0.16.0; RRID:SCR_018926) [106,107] (seqkit grep --invert-match -r -p “^-+$”). The resultant alignment was used to build an HMM using hmmbuild from the hmmer package (v3.2.1; RRID:SCR_005305) [30]. The combined GTDB and EukProt database was probed for matches to each domain HMM with the hmmsearch command (minimum *e*-value = 10^−3^) using the “wrap_hmmscan.pl” script produced by Dan Richter (available at https://github.com/MBL-Physiology-Bioinformatics/2021-Bioinformatics-Tutorial-Materials/tree/master/phylogenetics).

The top hit for each species was collected and realigned to the original HMM using hmmer hmmalign. Using a custom script to filter this new alignment, sequences with information-poor poly(K)/poly(Q) stretches >10 residues long or insertions that were not shared by 5% or more of other species were omitted. The remaining sequences were once again realigned using hmmalign, and a new HMM was built. This HMM was used to search the proteome database as before, this time using a stricter minimum *e*-value of 10^−4^ and retaining the top 3 hits of potential homologs. The *e*-values used were chosen based on previous analyses [108,109] to generate an initial pool of hits with a low false-negative rate, but potentially high false-positive rate as an input for further filtering.

For each protein, the domain-specific HMM search hits and original BLAST search hits were then compiled into one FASTA file and duplicate sequences removed with seqkit rmdup (--by-name). To validate that the putative homolog hits were most closely related to the proteins of interest, we performed a reciprocal BLAST against *S. cerevisiae*, *H. sapiens*, and *A. thaliana* proteomes. Hits with the greatest similarity (lowest *e*-value) to known annotations of the protein of interest were considered “putatively valid”. Hits that showed similarity to the protein of interest but a greater similarity to a different protein in the model species were considered “potentially valid”. If hits showed greater similarity to an unrelated protein known to bear a similar structure (e.g., TPR-containing protein Cyc8 shares similarities with Ctr9), they were blacklisted and filtered out. Hits that showed no significant similarity to the protein of interest were considered dubious. Potentially valid and dubious hits were excluded if they showed no significant similarity to the protein of interest by either NCBI BLAST (RRID:SCR_004870) or PFAM scans of the sequence (RRID:SCR_006695) [27]. The remaining sequences were aligned with the addition of outgroup sequences using MUSCLE (v5.1; RRID:SCR_011812) (-super5) and trimmed using ClipKIT (v1.4.1; RRID:SCR_026411) [110] (-m kpi-gappy). Phylogenetic trees were constructed with both FastTree (v2.1.10; RRID:SCR_015501) [111] (default settings) and iqtree2 (v2.2.2.7; RRID:SCR_017254) [112,113] (-m MFP -B 1000 --mem 250G -T AUTO). Outgroups and false-positive hits were manually removed to provide a final list of probable homologs. Manual removal was performed by inspection of domain architecture (predicted using PFAM scans) and gene trees to yield a final set of filtered orthologs. Since the S1 domain of Spt6 is common amongst RNA-binding proteins, HMM searches for the S1 domain were poorly selective for Spt6 and were thus excluded from the analysis. Blacklisted proteins and outgroup proteins are provided in Zenodo repository (see Data availability statement).

To search for putative TEF orthologs in Archaea, we performed scans with each finalized TEF domain HMM against GTDB Asgardarchaeota proteomes (minimum *e*-value = 10^−3^) and manually filtered hits as described above. For Metazoan or Fungi targeted searches, a BLAST search was performed using human and yeast orthologs against their respective databases (-max_target_seqs 50000) and only the hit with the lowest *e*-value for each species was retained, followed by alignment with MUSCLE (-super5). Filtered orthologs identified in the Eukprot/GTDB database search were aligned with MUSCLE (-super5) and a custom script was used to identify the mapping of analogous residues between *H. sapiens* and *S. cerevisiae* TEF reference sequences.

### Alternative search strategies for TEF homologs

To search for TEF homologs in specific sub-clades, a four-step approach was used. In clades where a given domain was not detected in any species by the HMM searches, FoldSeek was used to detect domains using structural similarity. FoldSeek searches were performed on the web server (RRID:SCR_027018) [33]. When available, existing crystal structures of domains were used as inputs for the search and retrieved from the Research Collaboratory for Structural Bioinformatics Protein Data Bank (https://www.rcsb.org/; RRID:SCR_012820) [114] - Rtf1-HMD (PDB ID 5E8B) [75], Cdc73-NTD (PDB ID 5YDE) [40], Ctr9-TPR motifs (PDB ID 6AF0) [44] and Rtf1-Plus3 (PBD ID 4L1P) [115]. In other cases, AlphaFold-predicted structures trimmed to the domain boundaries were used as inputs. Searches were performed against the AlphaFold/Uniprot50 v4 database by filtering for the clade indicated in the Zenodo repository (see S1 Table and Data availability statement). The remaining parameters were set to default. In cases where FoldSeek was unable to detect the domain of interest, AlphaFold3 web server [116] was used to predict the structures of candidate orthologs. Predicted structures were then manually inspected for the presence of the domain of interest. In cases where no protein hit corresponding to the protein of interest was identified by the HMM search, this was not carried out. When FoldSeek and AlphaFold3 were unable to detect the domain of interest, PSI–BLAST was performed. PSI–BLAST was performed on the BLAST web server (RRID:SCR_004870) [117] and was carried out using default parameters with the following exceptions: the Database was set to ClusteredNR, Organism was set to taxa indicated in S1 Table. Max target sequences was set to 20,000, and Expect threshold was set to 1. To maximize our chances of finding these domains using PSI–BLAST in the mentioned taxa, hits from closely related clades were used as templates. In clades where PSI–BLAST was unable to detect proteins of interest, additional proteomes corresponding to these clades were searched using custom-generated HMMs of TEF domains. Proteomes for clades indicated in S1 Table were downloaded from NCBI (March 13, 2026, see Data availability statement). We then scanned these proteomes using the finalized domain HMMs (minimum *e*-value = 10^−3^) and manually filtered hits. Manual filtration was conducted by inspection of domain architecture and AlphaFold3 predictions (see S1 Table and Data availability statement).

### Relative evolutionary rate calculations and structural representations

Each final set of homologs (EukProt + GTDB, refseq Metazoan, and refseq Fungi) was aligned using MAFFT (v7.471; RRID:SCR_011811) (--auto –anysymbol), then MSAs were trimmed using ClipKIT (v1.4.1; RRID:SCR_026411) (-m kpi-gappy). The trimmed MSAs were used to build trees using iqtree2 (v2.2.2.7; RRID:SCR_017254) (-m MFP -B 1000 --mem 250G -T AUTO). Separately, copies of the original MSA were processed such that columns not represented in the reference of interest (*H. sapiens* or *S. cerevisiae*) were discarded using a custom script. MEGA11 (RRID:SCR_000667) [118] was used to calculate relative evolutionary rates (RERs). To do so, iqtree2 TEF gene trees and MAFFT MSAs (condensed to columns represented in the *H. sapiens* or *S. cerevisiae* TEF reference as indicated in text) were used as input for the “Estimate Rate at Each Site (ML)” analysis (—Statistical Method “Maximum Likelihood”—Substitutions Type “Amino Acid”—Model/Method “JTT”— ates among sites “Gamma Distributed”—No. of Discrete Gamma Categories “5”). All sites were used for the calculation, and no branch swap filter was applied. RERs are calculated such that RER = 0 is invariant, RER = 1 is an average rate of evolution, and RER > 1 is a greater than average rate of evolution. Conservation scores were calculated as -log10(RER + 0.1). Final modified and unmodified MSAs, gene trees, RERs, and conservation scores are provided in Zenodo repository.

To map RERs onto molecular structures the ß-factors of the AlphaFold predictions of the relevant monomers were overwritten using a custom script. We have provided the associated sessions in the open-source ChimeraX viewer (v1.9; RRID:SCR_015872) [81] as supporting files in Zenodo repository (see Data availability statement). Per-residue surface-accessible surface area (SASA) and surface lipophilicity maps were calculated in ChimeraX. SASA measurements were based on each TEF’s monomeric AlphaFold predicted structure and residues with SASA > 50 Å^2^ were considered “solvent-exposed”.

### AlphaFold3 structure predictions

AlphaFold3 (RRID:SCR_028034) [116] was used with default settings and without energy minimization to predict the structure of putative TEF homologs (Figs 2B–2D and S10; S1 Table) and the *H. sapiens* Spt6 SID (S10A and S10B Fig) and to model the putative interaction interface between Rtf1 and Ctr9 (S11C–S11G Fig). *H. sapiens* Spt6S1 domain, along with a Zn^2+^ ion, was used as input for the prediction. *H. sapiens* and *S. cerevisiae* Rtf1-Ctr9 multimer predictions were carried out using 10 different seeds (10, 15, 500, 43, 58, 5697, 4327, 798, 111, 287), with each seed yielding 5 models. Structures were visualized in ChimeraX.

### S1 insert size distribution analysis

A custom script was used to calculate the size of SID-like insertions within known Spt6 orthologs. Briefly, the MUSCLE-aligned MSA of top-scoring Spt6 orthologs from the Eukprot/GTDB search was loaded into R using the msa package (v1.38.0) [119] and trimmed to the columns between *H. sapiens* residues 1,176–1,226. For each protein sequence, the number of non-gap characters was counted and plotted.

### ERC network analysis and visualization

ERC network for TEFs was generated as described previously with limited modifications (RRID:SCR_015669) [67]. Briefly, a *Z*-score cut-off of 3.5 was applied to identify factors that share a high ERC with each TEF (*PAF1* = 7, *CTR9* = 11, CDC73 = 7, *RTF1* = 26, *LEO1* = 7, *SPT4* = 12, *SPT5* = 17, *SPT6* = 33, *SPN1* = 3, *ELF1* = 4 genes passing threshold). Functions of genes were manually annotated using the Saccharomyces Genome Database (SGD; RRID:SCR_004694) [120].

To verify that this TEF ERC network represents relevant connections and was not random, the global clustering coefficient of this network was compared to that of 10,000 randomly sampled networks. Each sampled network contained one random gene corresponding to each TEF to act as a query node. The top *N* genes with the highest ERC values with a query node were selected where *N* is the number of genes for the assigned TEF that passed the *Z*-score cutoff (e.g., the genes with the top 7 ERC values were collected for query nodes corresponding to *PAF1*). The resultant networks each had the same total number of nodes and the same number of edges originating from each query node as the TEF ERC network in Fig 5.

### Software used for data visualization

Sina plots in S4C and S11E Figs were made using Prism (v10.5.0; RRID:SCR_002798). Other sina plots were generated using the geom_sina command in the ggforce package (v0.5.0; RRID:SCR_022575) [121]. UpSet plots were made in R using the ComplexUpSet package (v1.3.3; RRID:SCR_022752) [122]. Phylogenetic trees were visualized and annotated using iTOL (RRID:SCR_018174) [123]. Networks in Figs 3C and 5 were generated in Cytoscape (v3.10.2; RRID:SCR_003032) [124] and manually edited for visualization in Adobe Illustrator (RRID:SCR_010279). Multiple sequence alignment snapshots were rendered using the AliView software (v1.28; RRID:SCR_002780) [125].

## Supporting information

S1 Fig TEF homolog search strategy. Diagram describing pipeline for TEF homolog searches combined from EukProt and GTDB databases. See Materials and methods for more details. POI, Protein of interest; DOI, Domain of interest; HMM, Hidden Markov Model; Db, Database; MSA, Multiple Sequence Alignment.
(TIF)

S2 Fig Comparison of HMM vs. BLAST search efficacy for Paf1C subunits. **(A–E)** UpSet plots depicting the number of homologs collected using BLAST and domain-specific HMM searches for (A) Paf1, (B) Ctr9, (C) Cdc73, (D) Rtf1, and (E) Leo1. Plots reflect the source of each protein hit, not the presence of each domain. For example, 53 Paf1 orthologs were identified by searching EukProt and GTDB proteomes using an HMM built from the Paf1 Leo1-binding domain, but were not found when searching via the Paf1 Ctr9-binding domain or by BLAST with the human Paf1 sequence. This does not imply that in the 53 hits identified by searching for the Paf1 Leo1-binding domain, a Ctr9-binding domain was not detectable in the downstream analyses. Location of data files in the Zenodo repository used to generate plots in this figure has been provided in S3 Table.
(TIF)

S3 Fig Comparison of HMM vs. BLAST search efficacy for other TEFs. **(A–E)** UpSet plots depicting the number of homologs collected using BLAST and domain-specific HMM searches for (A) Spt4, (B) Elf1, (C) Spn1, (D) Spt5, and (E) Spt6. Plots reflect the source of each protein hit, not the presence of each domain. See S2 Fig for details. Location of data files in the Zenodo repository used to generate plots in this figure has been provided in S3 Table.
(TIF)

S4 Fig Detection of domains is dependent on the completeness of the source proteomes and the information content of the HMM. **(A)** Stacked bar charts indicating the percentage of proteomes in which the domains were detected, grouped by the source of the predicted proteome. Fisher’s Exact Test with multiple comparisons correction (Benjamini–Hochberg) was used to test if the probability of detecting a domain from genome-derived or transcriptome-derived proteomes is significantly different. **(B)** Sina plots comparing the distributions of Benchmarking Universal Single-Copy Ortholog (BUSCO) Completeness [126] of EukProt proteomes in which the indicated domain was detected versus not. Wilcoxon rank-sum test with multiple comparisons correction (Benjamini–Hochberg) was used to determine if the distribution of BUSCO proteome completeness scores is significantly correlated with domain detection across eukaryotic species. **(C)** Sina plot showing the distributions of CheckM2 genome completeness estimates of GTDB prokaryotes examined in this study (CheckM2 completeness >70% is “Substantially Complete”) [127,128]. Left—Distribution of CheckM2 estimates used in the 304 species search. Right—Distribution of CheckM2 estimates used in the expanded Asgard archaea search. **(D)** Scatter plot depicting the relationship between the number of species in which a domain was detected and the information in the HMM. The ratio between p relE (mean positional relative entropy, in bits) and EFFN (effective sequence number) was used as a measure of HMM information content [129]. Spearman’s correlation was used to determine the relationship between the detection of the domain and information in the HMM. *ρ*-value indicates a positive correlation between the information in the HMM and detection of the domain. To further characterize the relationship between detectability of domains and the information in the HMMs, the data were fit to a linear regression model (*y* = *ß*_0_ + *ß*_1_ × log(*x*)). Band represents 95% confidence interval. **(E)** Bar plot indicating percentage of Asgardarchaeota species (*n* = 218) in which Spt5, Spt4, and Elf1 orthologs were detected. See Materials and methods for more details. *p < 0.05; **p < 0.01; ***p < 0.001. Location of data files in the Zenodo repository used to generate plots in this figure has been provided in S3 Table.
(TIF)

S5 Fig Species-level variation in detection of TEF domains. Expanded tree from Fig 1 depicting the detection status of TEF domains in each species in the combined EukProt/GTDB database. Each column corresponds to a domain in the indicated protein (listed at bottom), and each row corresponds to a proteome from an organism. A filled box indicates that the domain was detected in the proteome of the organism. Colored bars on the right represent clades. Location of data files in the Zenodo repository used to generate this figure has been provided in S3 Table.
(TIF)

S6 FigOdds of detecting Paf1C domains are lower in some clades.**(A)** Heatmap showing log_2_(Odds Ratio) of domain detection in different clades. The Odds Ratio was calculated as follows: let C be a clade of interest and D be a domain of interest. Let *x* be the number of species in clade C in which domain D was detected. Let *y* be the number of species in which domain D was detected in clades other than clade C. Let S_c_ be the total number of species in clade C. In total, our dataset contained proteomes from 227 eukaryotes. Therefore, for each domain-clade pair, an Odds Ratio was calculated as: $Odds\;Ratio=\;\frac{\frac{x}{S_{c}-x}}{\frac{y}{(\text{2}\text{2}\text{7}-S_{c})-y}}$. Bar plot on the right indicates number of species analyzed in each clade. Red arrows indicate clades in which the Odds Ratio for the detection of some Paf1C domains was less than 1. **(B)** Sina plots comparing the distributions of BUSCO completeness scores of EukProt proteomes from different clades. Red line and value indicate median BUSCO completeness score of all EukProt proteomes analyzed. Wilcoxon rank-sum test with multiple comparisons correction (Benjamini–Hochberg) was used to determine if the distribution of scores from proteomes in a clade significantly differ from the distribution of all proteomes in the database. *p < 0.05; **p < 0.01; ***p < 0.001. Location of data files in the Zenodo repository used to generate plots in this figure has been provided in S3 Table.(TIF)

S7 Fig Multi-domain architectures of TEFs are broadly conserved. **(A, B)** UpSet plots depicting the coincidence of domain detection in homologs of indicated proteins as determined by HMMER hmmscan using custom HMMs. Scan domain eValue (--domE) threshold set to 10^−3^ [30]. Location of data files in the Zenodo repository used to generate plots in this figure has been provided in S3 Table.
(TIF)

S8 Fig Conservation score analysis of TEF residues. **(A)** Conservation scores of Ctr9, Leo1, Spt5, Spn1, Paf1, Elf1, and Spt4 across each residue along the *H. sapiens* homolog. See Fig 3 legend for a description of the plots. In the Spn1 line plot, gaps indicate residues that are unique to the human Spn1 homolog. **(B)** Sina plot showing the distribution of conservation scores of TEF residues. Data points have been colored by quintile. Dashed line indicates the cut-off used for residues considered as slowly evolving in Fig 3B. **(C)** Diagram of pairwise interfaces in the transcription elongation complex (PDB: 9EH2). Number of residues of each TEF (y-axis/Interactor-A) within 4 angstroms of other components of the elongation complex (x-axis/Interactor-B). Color intensity scales with the number in each tile. Location of data files in the Zenodo repository used to generate plots in this figure has been provided in S3 Table.
(TIF)

S9 Fig TEF detection and sequence conservation in metazoan and fungal proteome scans. **(A)** Percentage of proteomes from RefSeq Fungi (*n* = 128) and RefSeq Metazoa (*n* = 706) databases for which a homolog was identified by BLAST search. **(B–D)** Different classes of conserved residues in TEFs, highlighted on the AlphaFold2 predicted structures of the human homologs. The relative positions of select additional factors on the transcription elongation complex are depicted as cartoon diagrams for clarity. Residues that are unaligned between the *S. cerevisiae* and *H. sapiens* orthologs and are not well conserved have been colored black. (B) Concordantly conserved residues (top 20% conserved residues in metazoan and fungal homologs) in the IWS1 domain of Spn1 (left) and the HMD of Rtf1 (right). Residues in Spn1 that are predicted to extend close to where Elf1 and Spt5 bind to elongating RNAPII are Arg751, Ala752, Val754, Tyr762, Arg765, and Pro766. Residues in Rtf1 that are conserved in both clades and interact with Spt16 are His263, Arg267, Ala281, Leu285, Ala287, and Arg289. (C) Differentially conserved residues (top 20%–40% conserved residues in metazoan homologs and bottom 20% conserved residues in fungal homologs) in an uncharacterized region in Paf1 (left) and the TPR motifs of Ctr9 (right). (D) Top 20%–40% conserved residues in metazoan homologs that are not mappable in the *S. cerevisiae* homolog, highlighted in understudied regions in Cdc73 (left) and Rtf1 (right). Location of data files in the Zenodo repository used to generate plots in this figure has been provided in S3 Table.
(TIF)

S10 Fig TEF AlphaFold2 Monomer Predictions Colored by pLDDT. AlphaFold2 predicted structures of *H. sapiens* TEFs indicated in Figs 4 and S9 colored by pLDDT scores of residues.
(TIF)

S11 Fig The S1 Insertion Domain is unique to metazoan and choanoflagellate Spt6 and is predicted to adopt a zinc-finger fold. **(A)** Left—AlphaFold3 prediction of the human Spt6 S1 insertion domain (SID) with Zn^2+^. Residues shown as sticks are predicted to coordinate a zinc ion. Right—AlphaFold3 prediction colored by pLDDT score. **(B)** Charge distribution of the human Spt6 S1 domain and SID surface residues displayed using the ChimeraX ‘coulombic’ command. **(C)** MSA snapshot of top-scoring Spt6 orthologs from eukaryotes, highlighting the insertion in Spt6 homologs in Metazoa and Choanoflagellata. MSA was trimmed using clipkit (--kpi-gappy) for clarity. **(D)** Sina plots showing the distribution of lengths of inserts in the S1 domain in Metazoa, Choanogflagellata, and other eukaryotic clades. MSA in panel C was examined to count the number of residues between positions aligning to the start and stop of the *H. sapiens* SID (residues 1,176–1,226). See Materials and methods for more details. Location of data files in the Zenodo repository used to generate plots in this figure has been provided in S3 Table.
(TIF)

S12 Fig Human Rtf1 harbors a region homologous to fungal Rtf1 Hook. **(A, B)** MSA of Rtf1 homologs from EukProt/GTDB (A) and RefSeq fungal proteome (B) searches. Boundaries of the Rtf1 Hook are bracketed in red. Hydrophobic residues predicted to interact with a lipophilic surface in Ctr9 are labeled with black boxes. Regions corresponding to Rtf1 Hook from the indicated species were collected, and all gap columns were removed. (A) Alignment of Rtf1 Hook region from *Homo sapiens*, *Saccharomyces cerevisiae*, *Schizosaccharomyces pombe*, *Yarrowia lipolytica*, and *Aspergillus nudilans*. (B) Alignment of Rtf1 Hook region from *Saccharomyces cerevisiae*, *Schizosaccharomyces pombe*, *Yarrowia lipolytica*, *Aspergillus nudilans*, and *Komagatella phaffi*. **(C)** Predicted aligned error (PAE) plots of top *S. cerevisiae* and *H. sapiens* Ctr9-Rtf1 co-fold predictions. PAE plot images were made in ChimeraX. **(D)** AlphaBridge [130] plots for *S. cerevisiae* (top) and *H. sapiens* (bottom) Ctr9-Rtf1 co-folds, highlighting regions involved in interaction (black lines) between the two proteins. Inner rings represent the pLDDT score of each residue. Predictions for *S. cerevisiae* and *H. sapiens* Ctr9-Rtf1 were generated using the AlphaFold3 web server. The output generated from the web server was downloaded as a.zip file and uploaded to the AlphaBridge web server (default parameters) to generate figures. **(E)** Distribution of AlphaFold3 Ranking Scores (*n* = 50) for indicated protein pairs. Dotted line represents median of the distribution, and the brown point indicates the top-scoring model. The analysis in the following panels was done using this top-scoring model. **(F)** Rtf1 Hook in complex with Ctr9 as predicted by AlphaFold3 for *H. sapiens* (yellow) and *S. cerevisiae* (green) homologs, aligned to homologous cryo-EM structure of *Komagatella phaffi* proteins (PDB: 7XSX). **(G)** Lipophilicity maps of *H. sapiens* and *S. cerevisiae* Rtf1 Hook and Ctr9 grooves as calculated using the ChimeraX molecular lipophilicity potential (mlp) command. Black asterixis indicate hydrophobic residues highlighted in panels A and B. Location of data files in the Zenodo repository used to generate plots in this figure has been provided in S3 Table.
(TIF)

S13 Fig Global clustering coefficient of the TEF ERC network is greater than that of 10,000 randomly generated networks. **(A)** Histogram showing the distribution of the global clustering coefficients of the 10,000 randomly sampled networks (see Materials and methods). The red line represents the coefficient of the ERC network in Fig 5 (0.115). **(B)** Stacked bar plot showing the number of genes above *Z*-score ≥3.5 threshold connected to each TEF in the ERC network. Green bars represent the number of unique genes connected to the TEF in the network. Location of data files in the Zenodo repository used to generate plots in this figure has been provided in S3 Table.
(TIF)

S1 Table Summary of alternative searches for domains in Discoba, Metamonada, and Alveolata. (XLSX)

S2 Table Location of data files in Zenodo repository used to generate plots in the main figures. (XLSX)

S3 Table Location of data files in Zenodo repository used to generate plots in the supplementary figures. (XLSX)

S1 Text Abbreviations. (TXT)

## Abbreviations

ERC: evolutionary rate covariation
HMMs: hidden Markov models
LECA: Last Eukaryotic Common Ancestor
MSAs: multiple sequence alignments
RERs: relative evolutionary rates
RNAPII: RNA Polymerase II
SID: S1 Insertion Domain
TEFs: Transcription elongation factors
