# Supplementary figures and images for: Evolutionary analysis of transcription elongation factors reveals conserved and lineage-specific regulatory domains
Source: PLoS Biol. 2026 Jun 15;24(6):e3003855. doi: 10.1371/journal.pbio.3003855 (PMC13289920; doi:10.1371/journal.pbio.3003855)

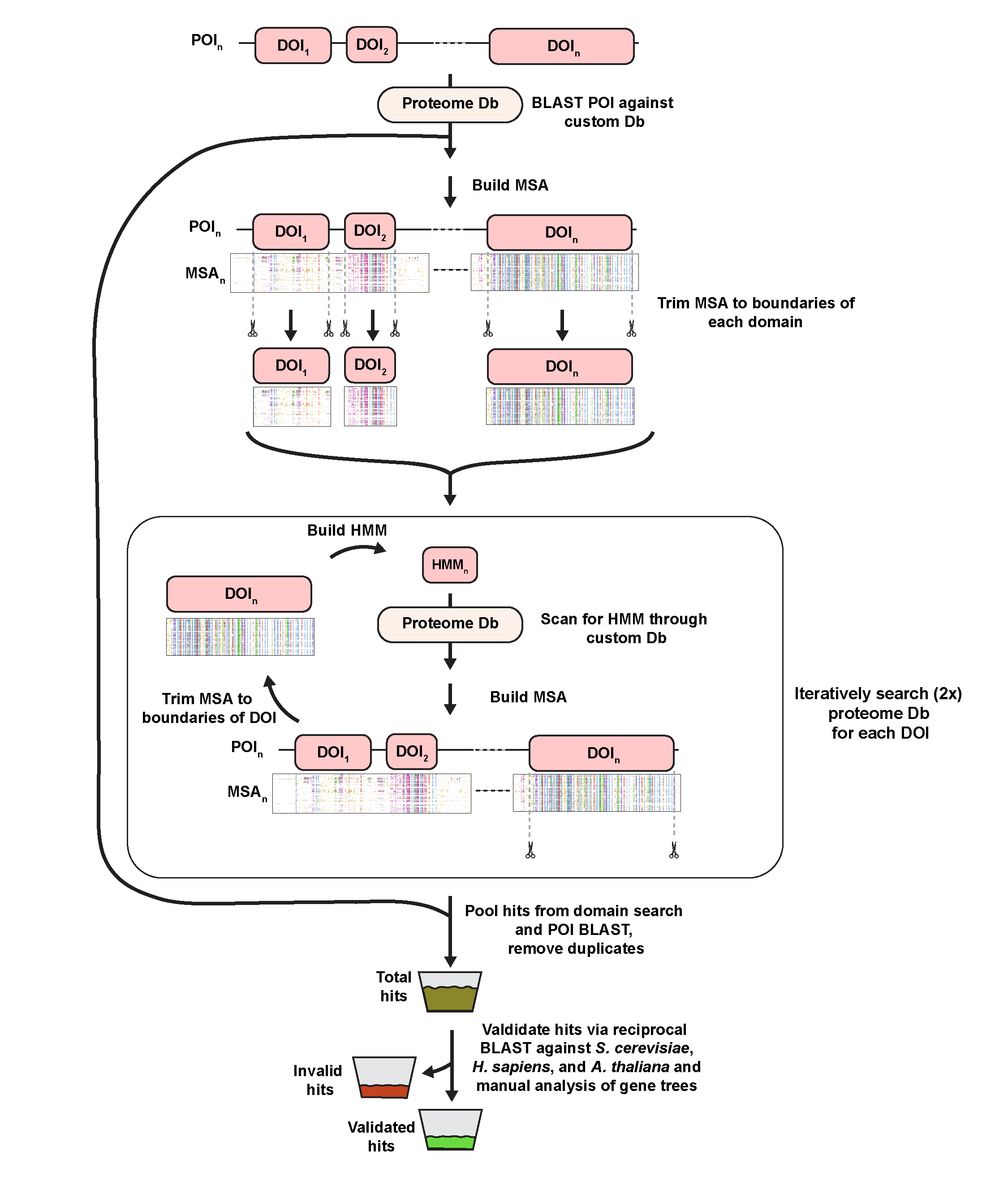

Supplement: S1 Fig — Diagram describing pipeline for TEF homolog searches combined from EukProt and GTDB databases. See Materials and methods for more details. POI, Protein of interest; DOI, Domain of interest; HMM, Hidden Markov Model; Db, Database; MSA, Multiple Sequence Alignment. (TIF) [file pbio.3003855.s001.tif]

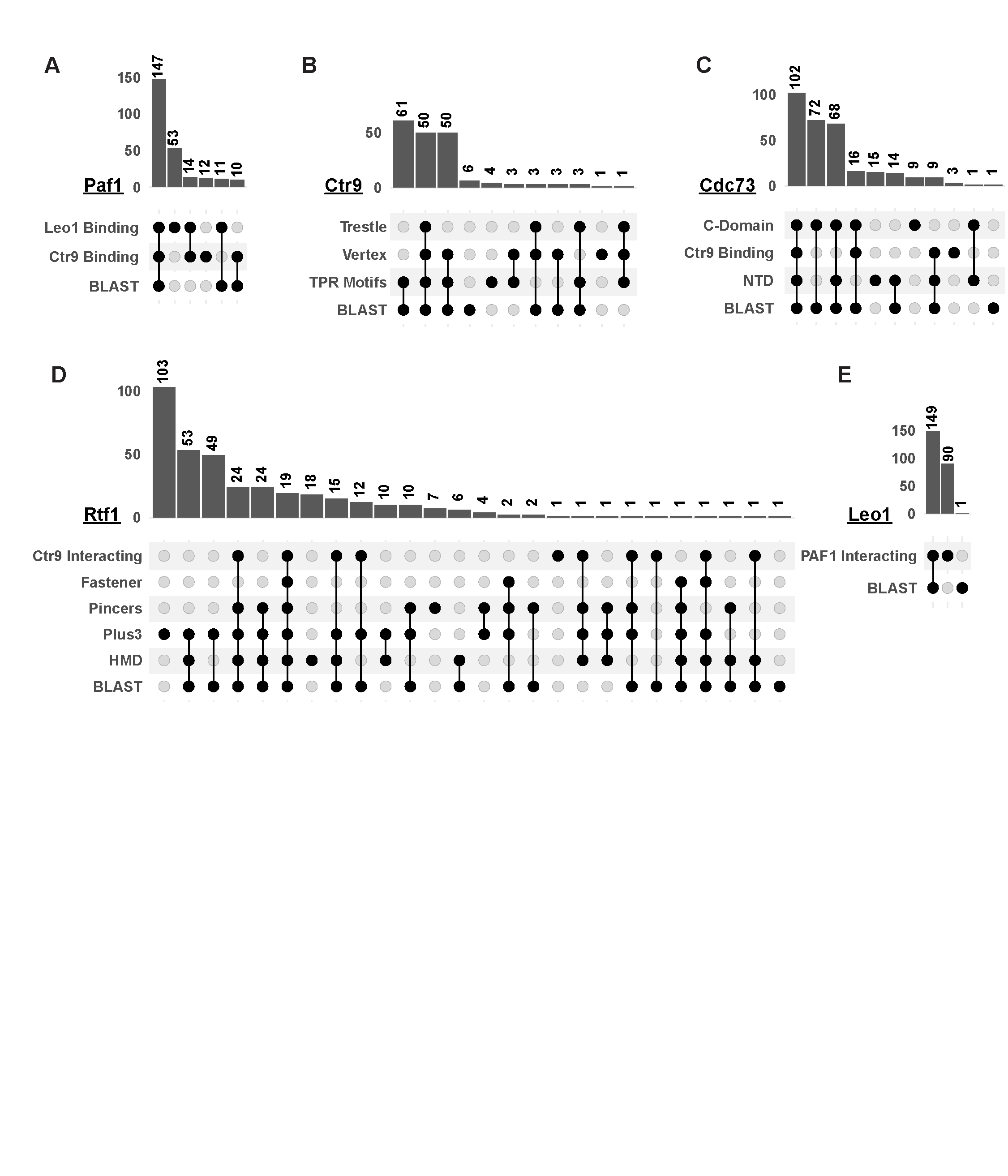

Supplement: S2 Fig — (A–E) UpSet plots depicting the number of homologs collected using BLAST and domain-specific HMM searches for (A) Paf1, (B) Ctr9, (C) Cdc73, (D) Rtf1, and (E) Leo1. Plots reflect the source of each protein hit, not the presence of each domain. For example, 53 Paf1 orthologs were identified by searching EukProt and GTDB proteomes using an HMM built from the Paf1 Leo1-binding domain, but were not found when searching via the Paf1 Ctr9-binding domain or by BLAST with the human Paf1 sequence. This does not imply that in the 53 hits identified by searching for the Paf1 Leo1-binding domain, a Ctr9-binding domain was not detectable in the downstream analyses. Location of data files in the Zenodo repository used to generate plots in this figure has been provided in S3 Table. (TIF) [file pbio.3003855.s002.tif]

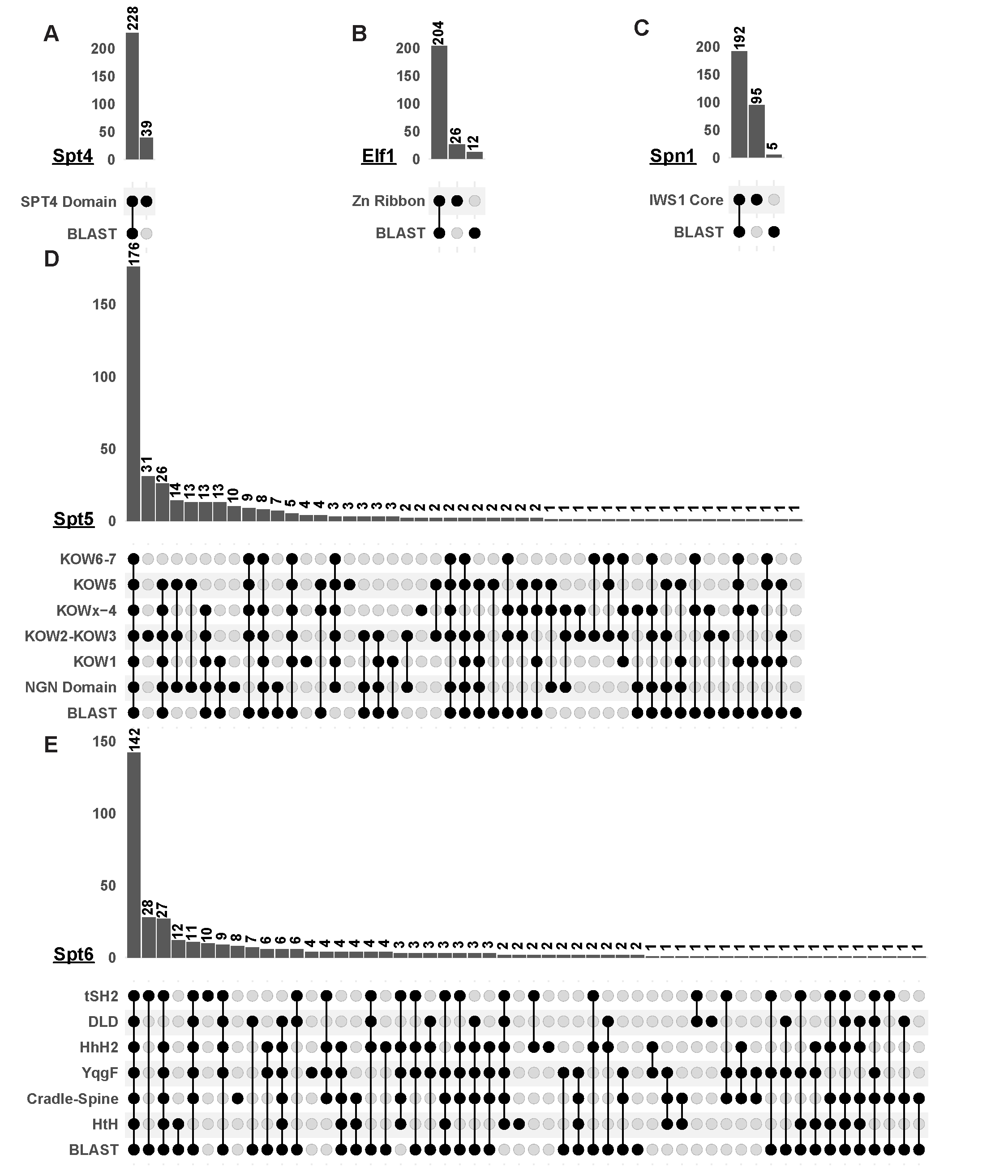

Supplement: S3 Fig — (A–E) UpSet plots depicting the number of homologs collected using BLAST and domain-specific HMM searches for (A) Spt4, (B) Elf1, (C) Spn1, (D) Spt5, and (E) Spt6. Plots reflect the source of each protein hit, not the presence of each domain. See S2 Fig for details. Location of data files in the Zenodo repository used to generate plots in this figure has been provided in S3 Table. (TIF) [file pbio.3003855.s003.tif]

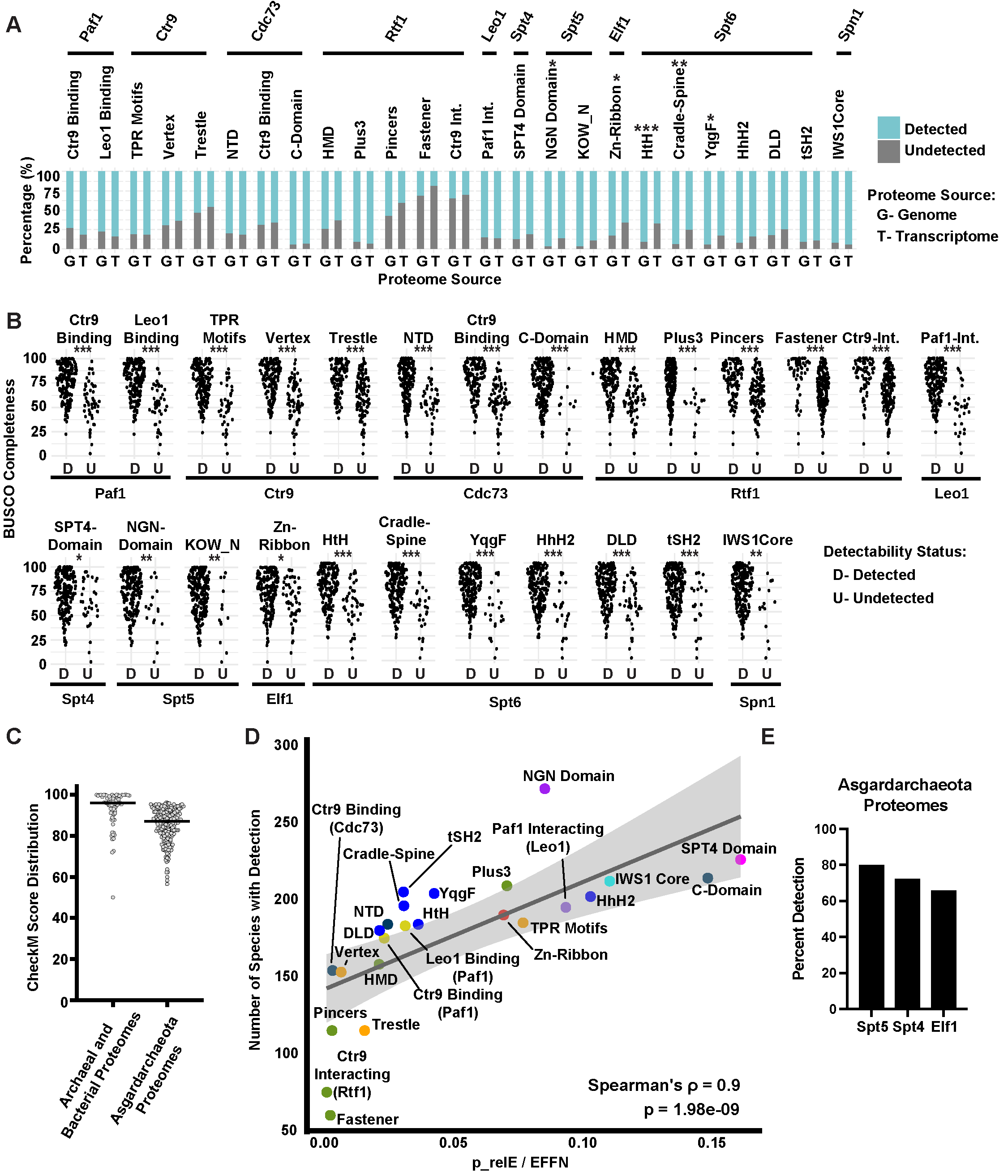

Supplement: S4 Fig — (A) Stacked bar charts indicating the percentage of proteomes in which the domains were detected, grouped by the source of the predicted proteome. Fisher’s Exact Test with multiple comparisons correction (Benjamini–Hochberg) was used to test if the probability of detecting a domain from genome-derived or transcriptome-derived proteomes is significantly different. (B) Sina plots comparing the distributions of Benchmarking Universal Single-Copy Ortholog (BUSCO) Completeness [126] of EukProt proteomes in which the indicated domain was detected versus not. Wilcoxon rank-sum test with multiple comparisons correction (Benjamini–Hochberg) was used to determine if the distribution of BUSCO proteome completeness scores is significantly correlated with domain detection across eukaryotic species. (C) Sina plot showing the distributions of CheckM2 genome completeness estimates of GTDB prokaryotes examined in this study (CheckM2 completeness >70% is “Substantially Complete”) [127,128]. Left—Distribution of CheckM2 estimates used in the 304 species search. Right—Distribution of CheckM2 estimates used in the expanded Asgard archaea search. (D) Scatter plot depicting the relationship between the number of species in which a domain was detected and the information in the HMM. The ratio between p relE (mean positional relative entropy, in bits) and EFFN (effective sequence number) was used as a measure of HMM information content [129]. Spearman’s correlation was used to determine the relationship between the detection of the domain and information in the HMM. ρ-value indicates a positive correlation between the information in the HMM and detection of the domain. To further characterize the relationship between detectability of domains and the information in the HMMs, the data were fit to a linear regression model (y = ß0 + ß1 × log(x)). Band represents 95% confidence interval. (E) Bar plot indicating percentage of Asgardarchaeota species (n = 218) in which Spt5, Spt4, and Elf1 orthol [file pbio.3003855.s004.tif]

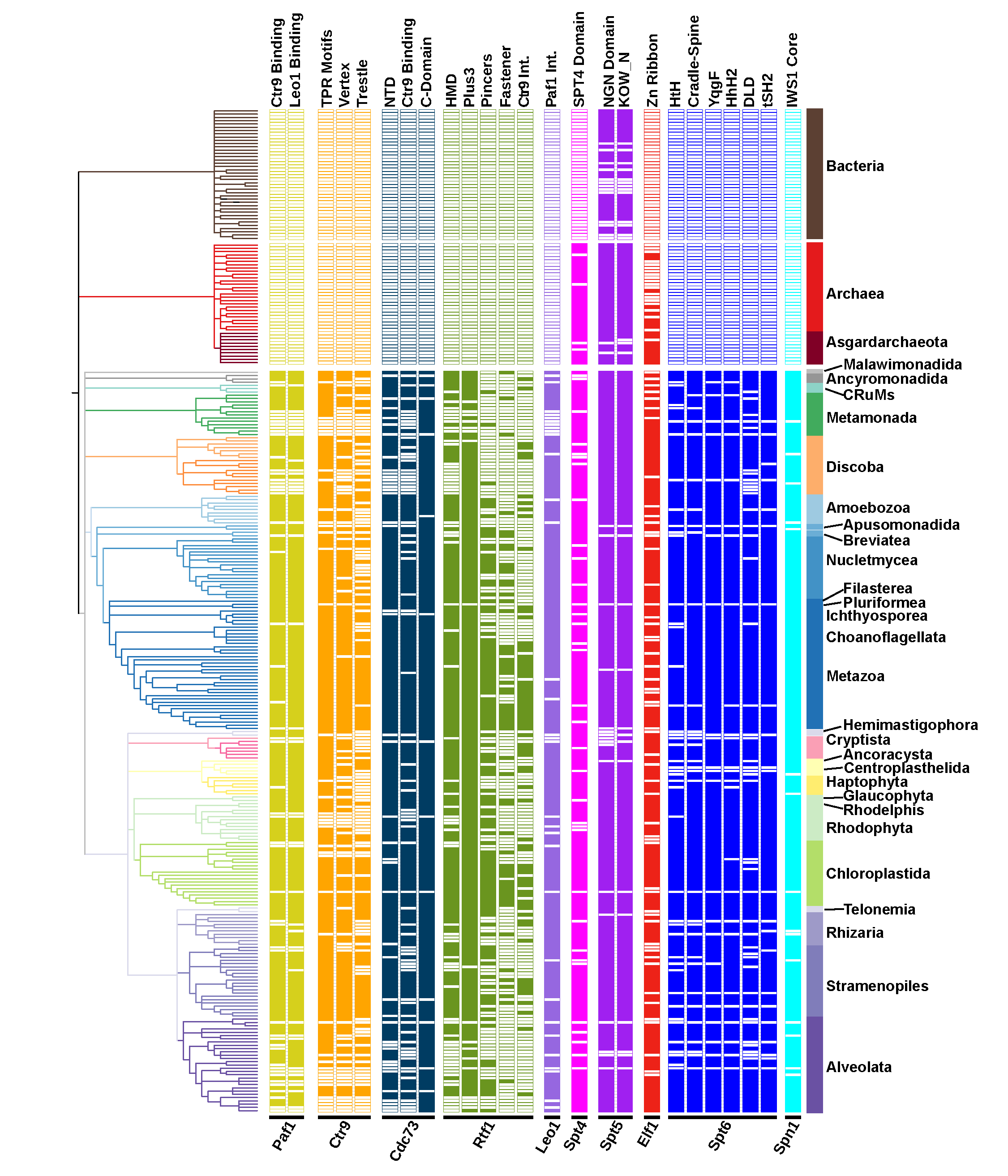

Supplement: S5 Fig — Expanded tree from Fig 1 depicting the detection status of TEF domains in each species in the combined EukProt/GTDB database. Each column corresponds to a domain in the indicated protein (listed at bottom), and each row corresponds to a proteome from an organism. A filled box indicates that the domain was detected in the proteome of the organism. Colored bars on the right represent clades. Location of data files in the Zenodo repository used to generate this figure has been provided in S3 Table. (TIF) [file pbio.3003855.s005.tif]

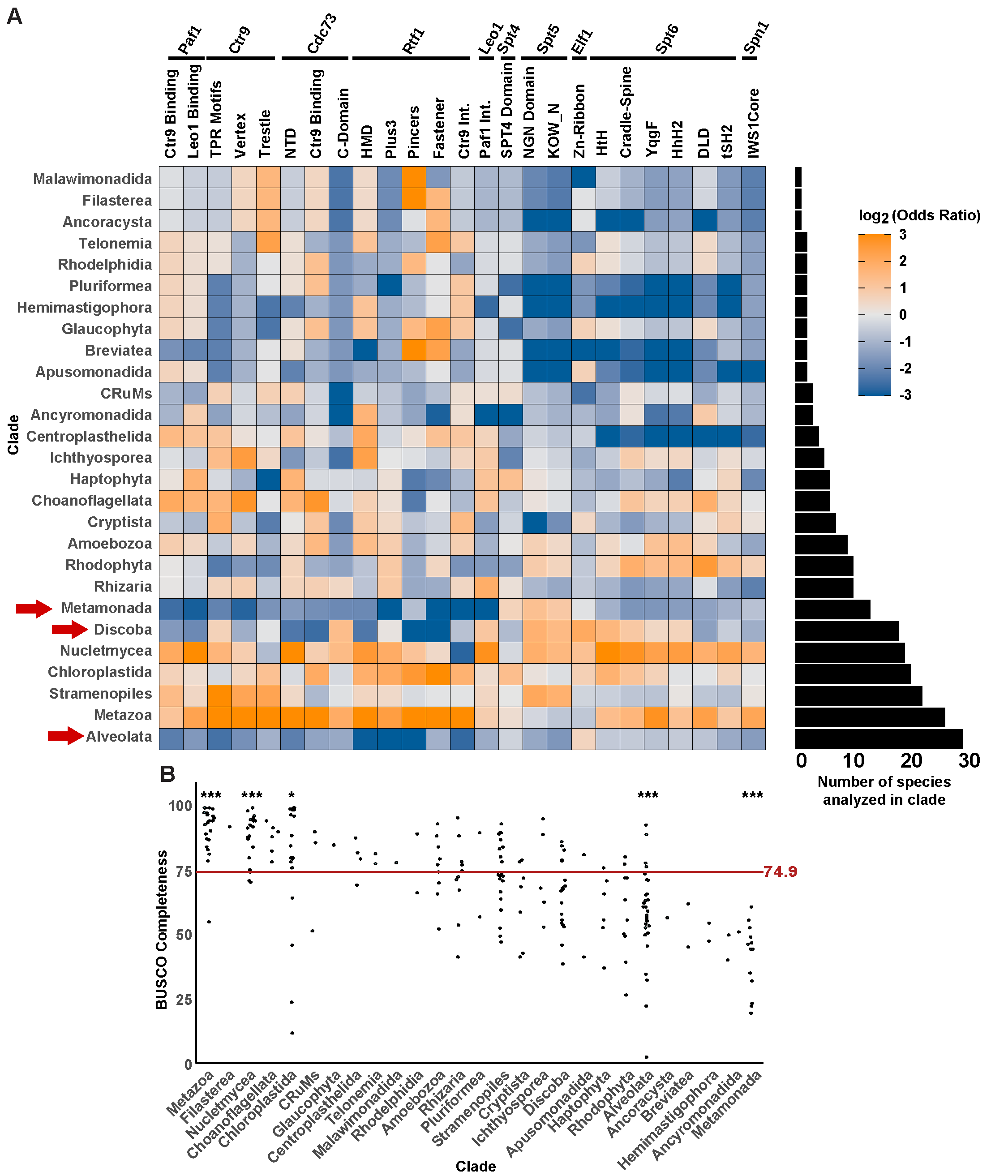

Supplement: S6 Fig — (A) Heatmap showing log2(Odds Ratio) of domain detection in different clades. The Odds Ratio was calculated as follows: let C be a clade of interest and D be a domain of interest. Let x be the number of species in clade C in which domain D was detected. Let y be the number of species in which domain D was detected in clades other than clade C. Let Sc be the total number of species in clade C. In total, our dataset contained proteomes from 227 eukaryotes. Therefore, for each domain-clade pair, an Odds Ratio was calculated as: Odds Ratio= xSc−xy(227−Sc)−y. Bar plot on the right indicates number of species analyzed in each clade. Red arrows indicate clades in which the Odds Ratio for the detection of some Paf1C domains was less than 1. (B) Sina plots comparing the distributions of BUSCO completeness scores of EukProt proteomes from different clades. Red line and value indicate median BUSCO completeness score of all EukProt proteomes analyzed. Wilcoxon rank-sum test with multiple comparisons correction (Benjamini–Hochberg) was used to determine if the distribution of scores from proteomes in a clade significantly differ from the distribution of all proteomes in the database. *p < 0.05; **p < 0.01; ***p < 0.001. Location of data files in the Zenodo repository used to generate plots in this figure has been provided in S3 Table. (TIF) [file pbio.3003855.s006.tif]

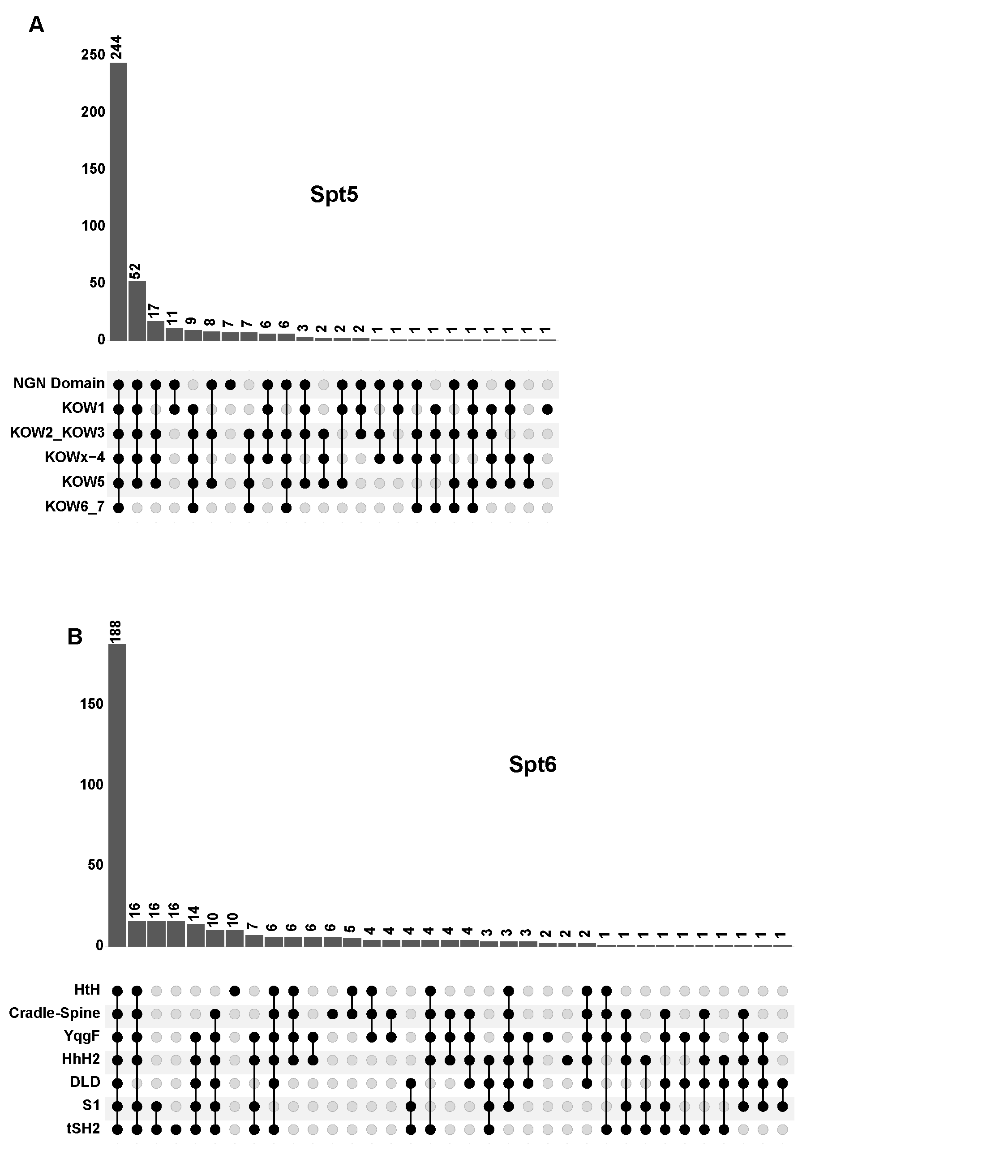

Supplement: S7 Fig — (A, B) UpSet plots depicting the coincidence of domain detection in homologs of indicated proteins as determined by HMMER hmmscan using custom HMMs. Scan domain eValue (--domE) threshold set to 10−3 [30]. Location of data files in the Zenodo repository used to generate plots in this figure has been provided in S3 Table. (TIF) [file pbio.3003855.s007.tif]

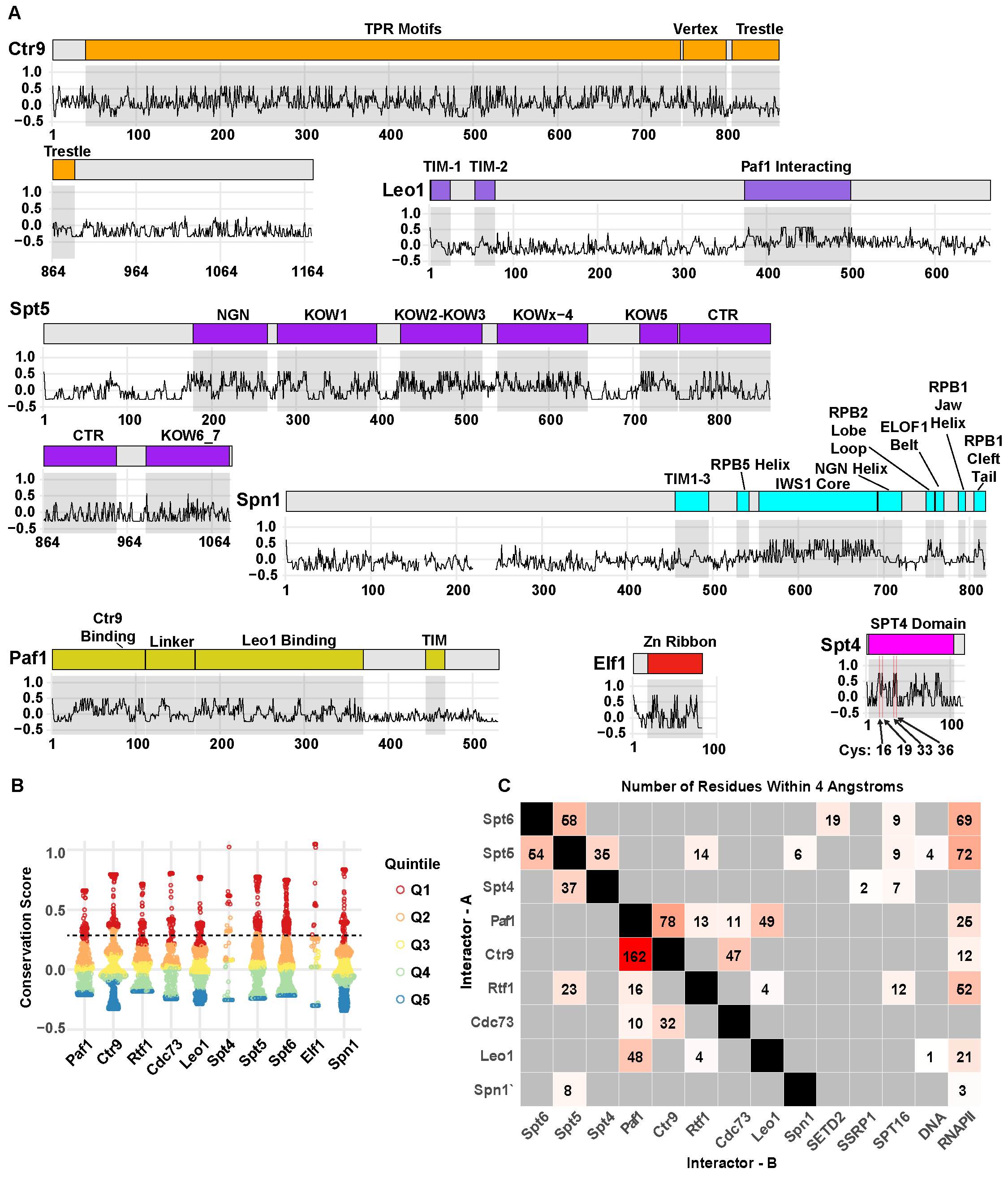

Supplement: S8 Fig — (A) Conservation scores of Ctr9, Leo1, Spt5, Spn1, Paf1, Elf1, and Spt4 across each residue along the H. sapiens homolog. See Fig 3 legend for a description of the plots. In the Spn1 line plot, gaps indicate residues that are unique to the human Spn1 homolog. (B) Sina plot showing the distribution of conservation scores of TEF residues. Data points have been colored by quintile. Dashed line indicates the cut-off used for residues considered as slowly evolving in Fig 3B. (C) Diagram of pairwise interfaces in the transcription elongation complex (PDB: 9EH2). Number of residues of each TEF (y-axis/Interactor-A) within 4 angstroms of other components of the elongation complex (x-axis/Interactor-B). Color intensity scales with the number in each tile. Location of data files in the Zenodo repository used to generate plots in this figure has been provided in S3 Table. (TIF) [file pbio.3003855.s008.tif]

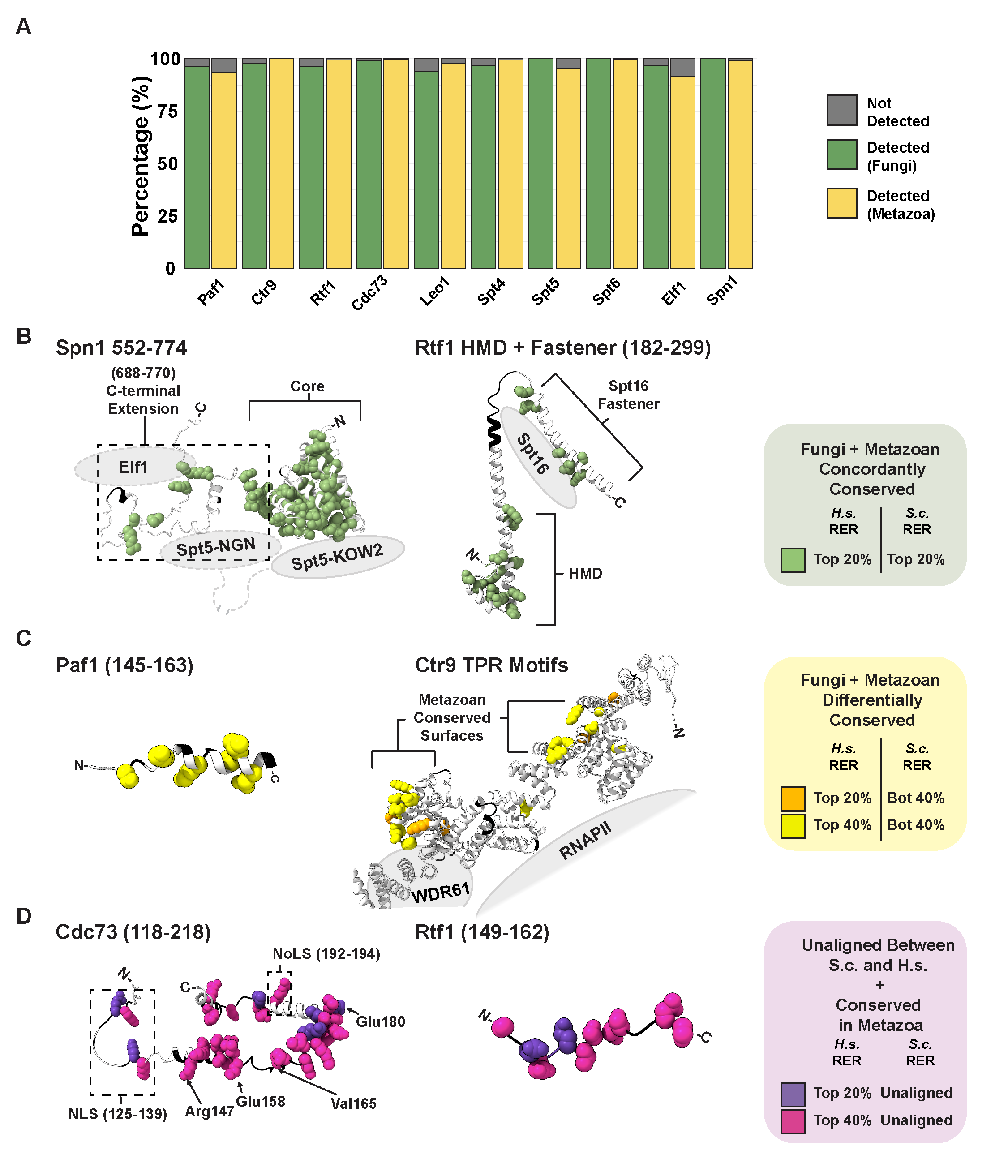

Supplement: S9 Fig — (A) Percentage of proteomes from RefSeq Fungi (n = 128) and RefSeq Metazoa (n = 706) databases for which a homolog was identified by BLAST search. (B–D) Different classes of conserved residues in TEFs, highlighted on the AlphaFold2 predicted structures of the human homologs. The relative positions of select additional factors on the transcription elongation complex are depicted as cartoon diagrams for clarity. Residues that are unaligned between the S. cerevisiae and H. sapiens orthologs and are not well conserved have been colored black. (B) Concordantly conserved residues (top 20% conserved residues in metazoan and fungal homologs) in the IWS1 domain of Spn1 (left) and the HMD of Rtf1 (right). Residues in Spn1 that are predicted to extend close to where Elf1 and Spt5 bind to elongating RNAPII are Arg751, Ala752, Val754, Tyr762, Arg765, and Pro766. Residues in Rtf1 that are conserved in both clades and interact with Spt16 are His263, Arg267, Ala281, Leu285, Ala287, and Arg289. (C) Differentially conserved residues (top 20%–40% conserved residues in metazoan homologs and bottom 20% conserved residues in fungal homologs) in an uncharacterized region in Paf1 (left) and the TPR motifs of Ctr9 (right). (D) Top 20%–40% conserved residues in metazoan homologs that are not mappable in the S. cerevisiae homolog, highlighted in understudied regions in Cdc73 (left) and Rtf1 (right). Location of data files in the Zenodo repository used to generate plots in this figure has been provided in S3 Table. (TIF) [file pbio.3003855.s009.tif]

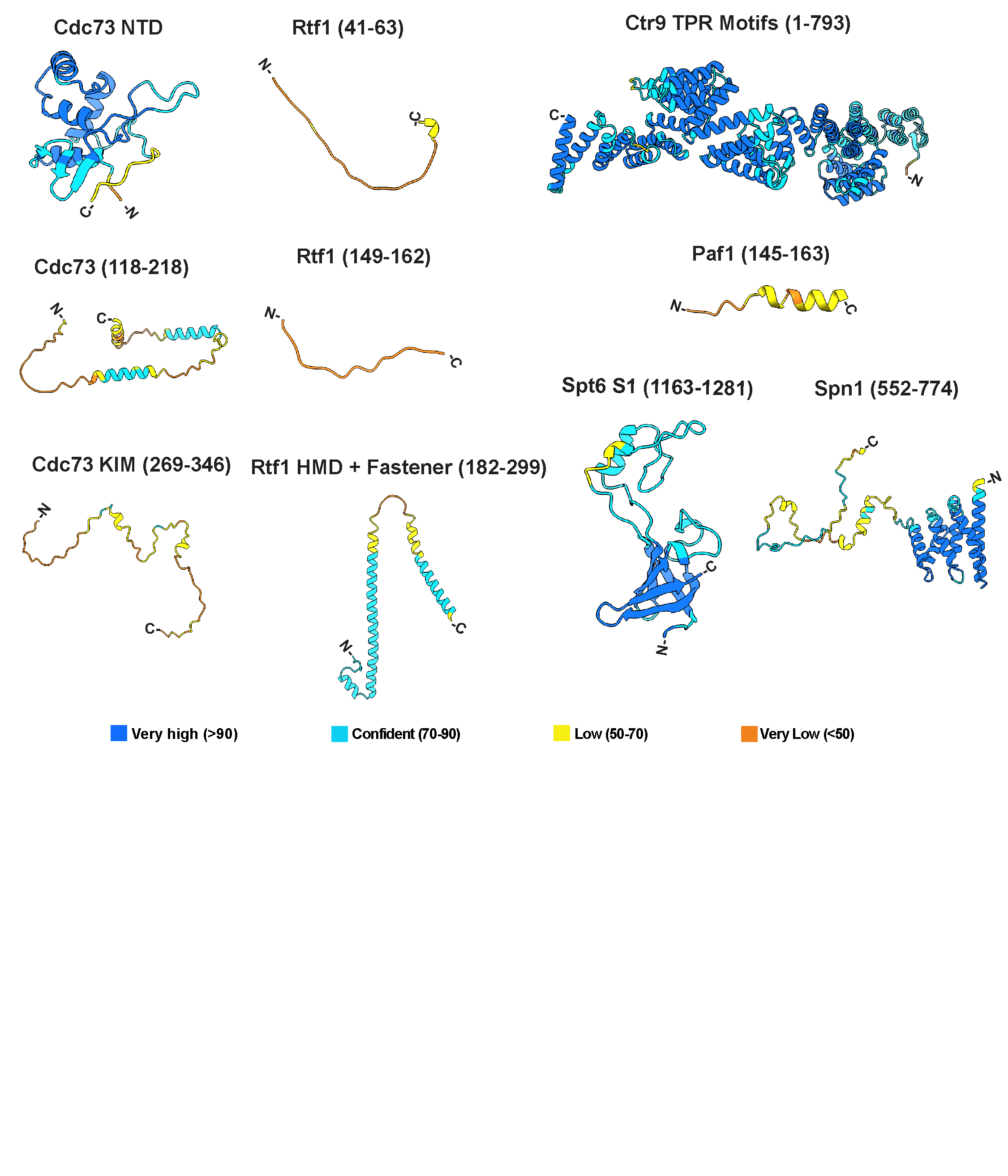

Supplement: S10 Fig — AlphaFold2 predicted structures of H. sapiens TEFs indicated in Figs 4 and S9 colored by pLDDT scores of residues. (TIF) [file pbio.3003855.s010.tif]

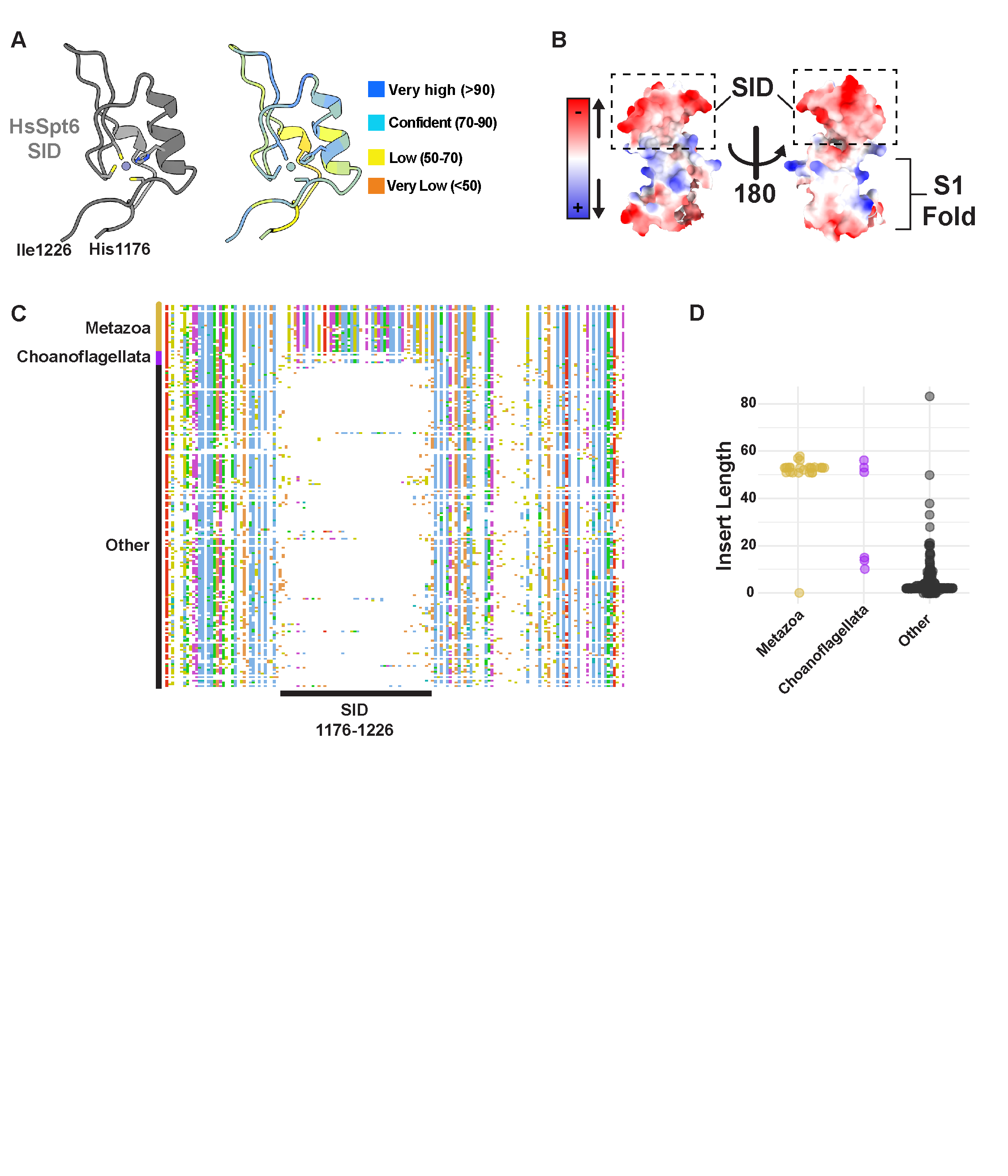

Supplement: S11 Fig — (A) Left—AlphaFold3 prediction of the human Spt6 S1 insertion domain (SID) with Zn2+. Residues shown as sticks are predicted to coordinate a zinc ion. Right—AlphaFold3 prediction colored by pLDDT score. (B) Charge distribution of the human Spt6 S1 domain and SID surface residues displayed using the ChimeraX ‘coulombic’ command. (C) MSA snapshot of top-scoring Spt6 orthologs from eukaryotes, highlighting the insertion in Spt6 homologs in Metazoa and Choanoflagellata. MSA was trimmed using clipkit (--kpi-gappy) for clarity. (D) Sina plots showing the distribution of lengths of inserts in the S1 domain in Metazoa, Choanogflagellata, and other eukaryotic clades. MSA in panel C was examined to count the number of residues between positions aligning to the start and stop of the H. sapiens SID (residues 1,176–1,226). See Materials and methods for more details. Location of data files in the Zenodo repository used to generate plots in this figure has been provided in S3 Table. (TIF) [file pbio.3003855.s011.tif]

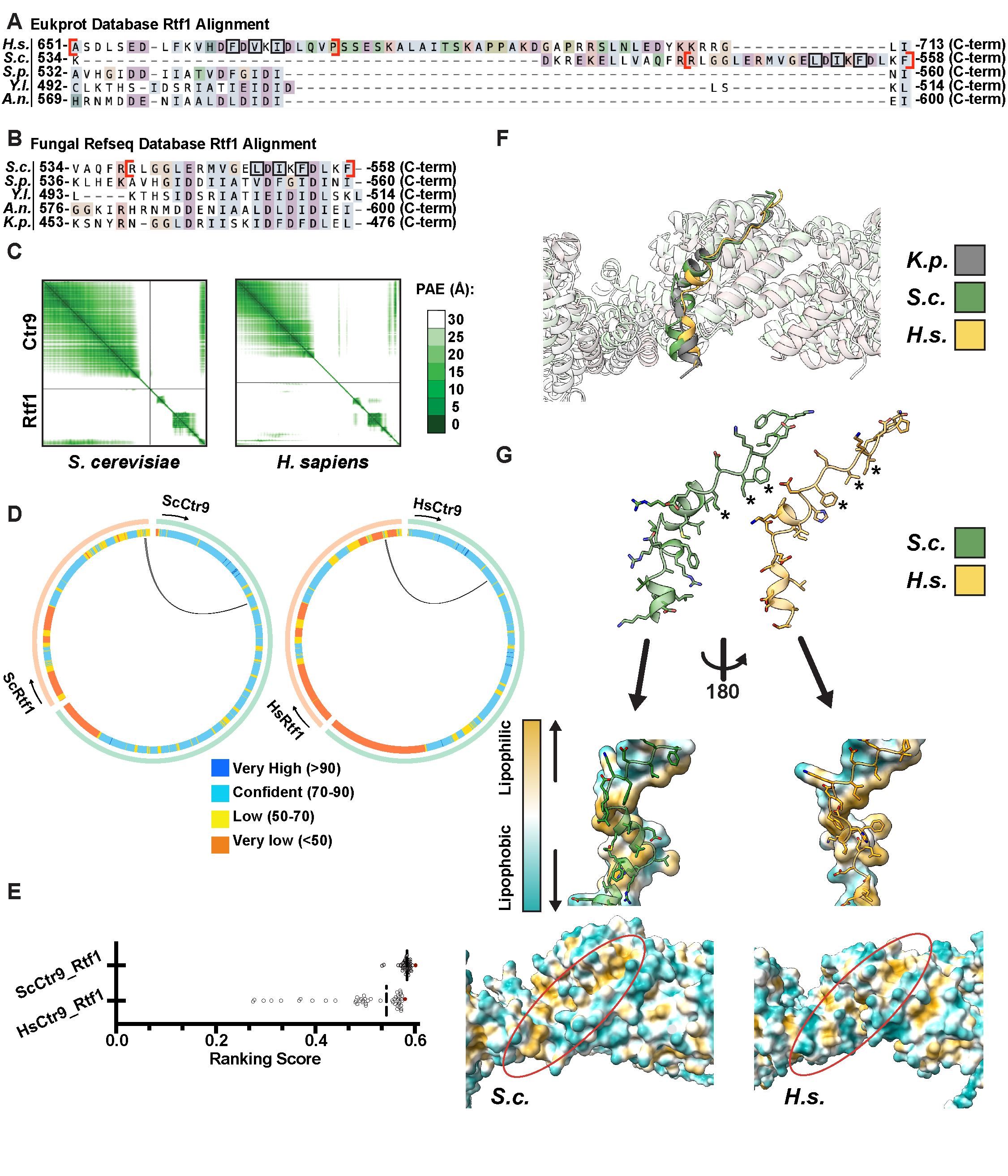

Supplement: S12 Fig — (A, B) MSA of Rtf1 homologs from EukProt/GTDB (A) and RefSeq fungal proteome (B) searches. Boundaries of the Rtf1 Hook are bracketed in red. Hydrophobic residues predicted to interact with a lipophilic surface in Ctr9 are labeled with black boxes. Regions corresponding to Rtf1 Hook from the indicated species were collected, and all gap columns were removed. (A) Alignment of Rtf1 Hook region from Homo sapiens, Saccharomyces cerevisiae, Schizosaccharomyces pombe, Yarrowia lipolytica, and Aspergillus nudilans. (B) Alignment of Rtf1 Hook region from Saccharomyces cerevisiae, Schizosaccharomyces pombe, Yarrowia lipolytica, Aspergillus nudilans, and Komagatella phaffi. (C) Predicted aligned error (PAE) plots of top S. cerevisiae and H. sapiens Ctr9-Rtf1 co-fold predictions. PAE plot images were made in ChimeraX. (D) AlphaBridge [130] plots for S. cerevisiae (top) and H. sapiens (bottom) Ctr9-Rtf1 co-folds, highlighting regions involved in interaction (black lines) between the two proteins. Inner rings represent the pLDDT score of each residue. Predictions for S. cerevisiae and H. sapiens Ctr9-Rtf1 were generated using the AlphaFold3 web server. The output generated from the web server was downloaded as a.zip file and uploaded to the AlphaBridge web server (default parameters) to generate figures. (E) Distribution of AlphaFold3 Ranking Scores (n = 50) for indicated protein pairs. Dotted line represents median of the distribution, and the brown point indicates the top-scoring model. The analysis in the following panels was done using this top-scoring model. (F) Rtf1 Hook in complex with Ctr9 as predicted by AlphaFold3 for H. sapiens (yellow) and S. cerevisiae (green) homologs, aligned to homologous cryo-EM structure of Komagatella phaffi proteins (PDB: 7XSX). (G) Lipophilicity maps of H. sapiens and S. cerevisiae Rtf1 Hook and Ctr9 grooves as calculated using the ChimeraX molecular lipophilicity potential (mlp) command. Black asterixis indicate hydrophobic residues highligh [file pbio.3003855.s012.tif]

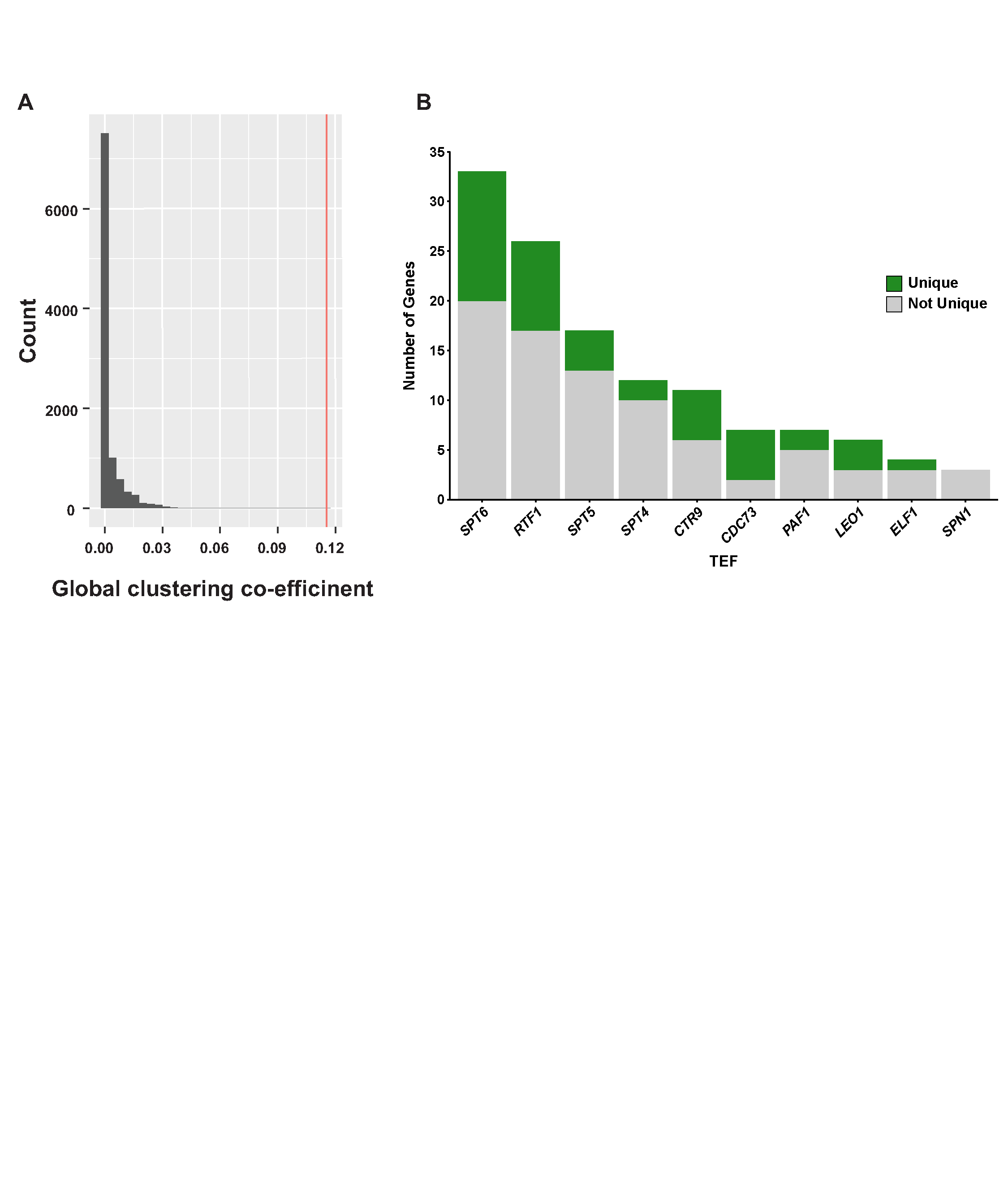

Supplement: S13 Fig — (A) Histogram showing the distribution of the global clustering coefficients of the 10,000 randomly sampled networks (see Materials and methods). The red line represents the coefficient of the ERC network in Fig 5 (0.115). (B) Stacked bar plot showing the number of genes above Z-score ≥3.5 threshold connected to each TEF in the ERC network. Green bars represent the number of unique genes connected to the TEF in the network. Location of data files in the Zenodo repository used to generate plots in this figure has been provided in S3 Table. (TIF) [file pbio.3003855.s013.tif]
