# Supplementary material for: Evolutionary analysis of transcription elongation factors reveals conserved and lineage-specific regulatory domains
Source: PLoS Biol. 2026 Jun 15;24(6):e3003855. doi: 10.1371/journal.pbio.3003855 (PMC13289920; doi:10.1371/journal.pbio.3003855)
Supplement: Attachment — Submitted filename: Francette_Grover_ResponseToReviewers.pdf [file pbio.3003855.s018.pdf]

Response to Reviewer Comments

PBIOLOGY-D-25-04145R1
Francette, Grover et al.
"Evolutionary conservation and innovations of RNA polymerase II transcription elongation factors"

We are grateful to both reviewers for their insightful, thorough, and positive comments.

In the following text, we respond to reviewer comments point-by-point. To specifically refer to figures within the rebuttal, we label them as "Figure R#" (*e.g.* Figure R1).
Author responses are in blue. Reviewer comments are in black.

REVIEWER #1

This manuscript investigates the evolutionary relationships between transcription elongation factors using domain centric method to identify homologs. The authors clearly explain their rational and approach, and make interesting observations about relative conservation and origin of transcription elongation factors. The manuscript makes some compelling observations, including a putative zinc binding domain on SPT6. Overall, the manuscript will be a useful resource for anyone studying transcription elongation and provides new avenues for research. I have a few comments the authors should address prior to publication.

-The authors omitted WDR61 from their analysis. WDR61 is not a component of yeast PAF1c, but there is a possibility that it is a member of PAF1c in other organisms outside of human. The way that WDR61 interacts with human CTR9 would preclude it from being in the SKI RNA surveillance complex. This suggests that WDR61 is able to participate in multiple complexes that are perhaps mutually exclusive. **It thus would be nice if the authors could use their approach to indicate whether WDR61 is a constitutive member of PAF1c** or **whether it only appears in select clades**. It is understood that this analysis may be challenging.

The similarity of WDR61 to the hundreds of other non-redundant WD40 proteins (Zou et al, 2016) increases the "noise" of false positive hits. Additionally, as the reviewer states, current structures indicate that WDR61 shares an interface with Paf1C and the SKI complex, which would be incompatible with its inclusion in both complexes simultaneously. This means that the conservation scores of residues at the WDR61:Paf1C/SKI complex interface are likely to integrate the selective pressures to maintain inclusion in either complex. This greatly complicates interpretation. To address the reviewer's comment, we have made an attempt to analyze WDR61 using our existing pipelines and an AlphaFold screening approach.

One route to elucidate whether WDR61 is a constitutive member of Paf1C in other species would be to identify a signature of conservation specific to WDR61 interacting with Paf1C. Despite the incompatibility for WDR61 to simultaneously occupy both complexes, it is possible that there would be residues of WDR61 that are important for the Ctr9 interaction that would be dispensable for an interaction with the SKI complex. However, the interaction interface of WDR61 is extensively shared between the SKI complex and Paf1C in humans in cryo-EM structures (**Figure R1A-B**). In fact, of the 47 residues in hWDR61 within 5 Å of Ctr9, 31 are also within 5 Å of the SKI complex (**Figure R1C**). If a portion of the 16 remaining residues specific to the WDR61:Ctr9 interaction had specialized roles promoting inclusion of WDR61 in Paf1C, we would expect that the conservation scores of these residues would be higher amongst metazoans compared to fungi, which presumably lack this pressure. Therefore, we performed

metazoan- and fungal-specific BLAST searches and calculated the conservation scores within each clade as performed in Figure 4 and Figure S9 to try to identify such a pattern. We find that these residues (colored pink in **Figure R1A**) are generally peripheral to the WDR61-Ctr9 interface and exhibit similar conservation score patterns in metazoans and fungi (**Figure R1D**). These observations suggest that these residues are not under specific selective pressure in metazoans and thus differential conservation is unlikely to be a strong predictor for WDR61 inclusion within Paf1C.

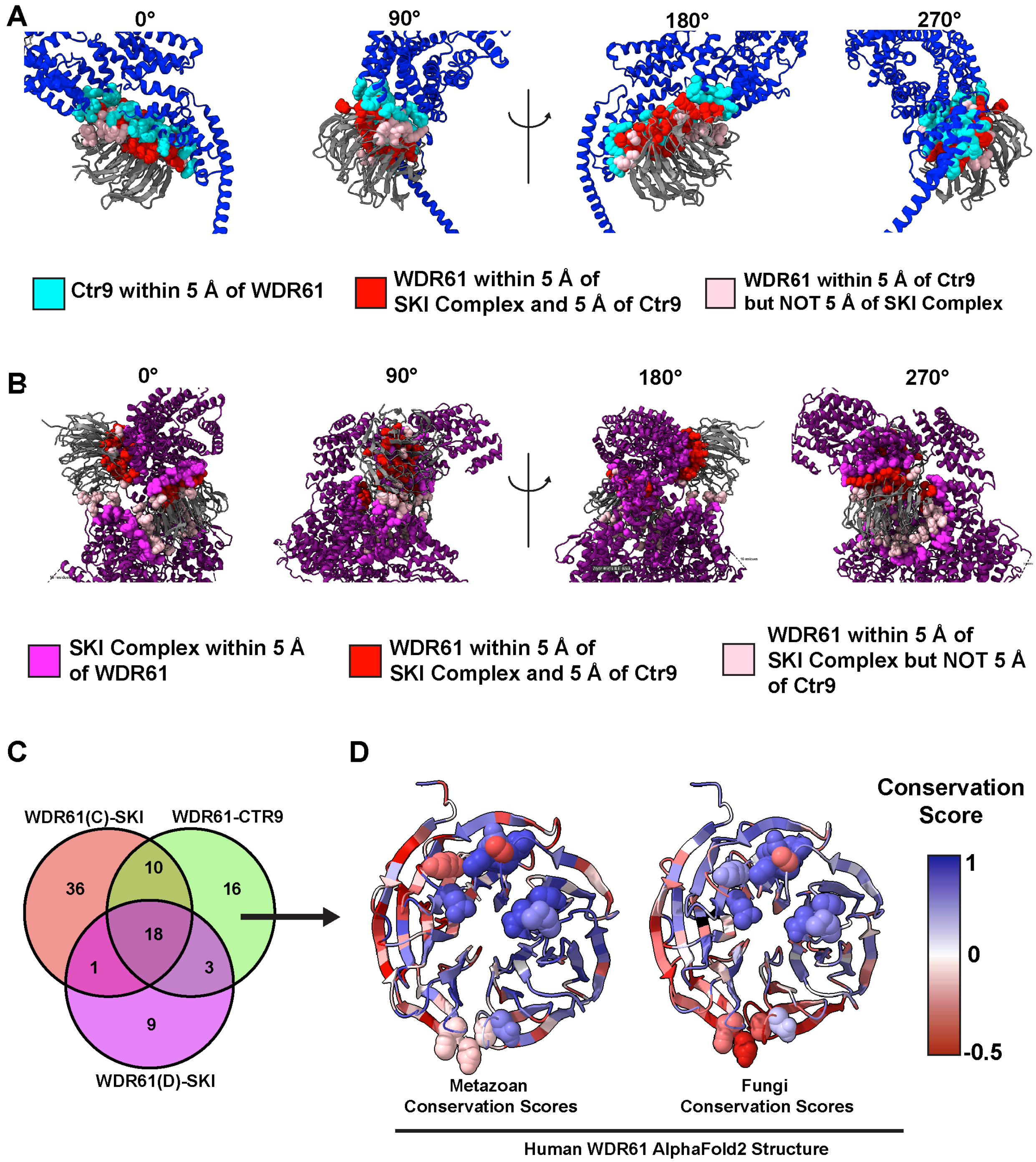

Figure R1. **Structural and conservation analysis of WDR61.** (A-B) Visualization of residues in WDR61 and interacting proteins in the human (A) activated transcription elongation complex (PDB:6TED; Vos *et. al.*, 2020) and (B) SKI complex (PDB: 7QDR; Kogel *et. al.*, 2022). In the structure, the SKI complex contains two WDR61 molecules (Chain C and Chain D in the PDB submission). The four views are progressive 90° rotations along the indicated axis. WDR61 is shown in gray. (C) Venn diagram depicting the number of WDR61 residues within 5 Å of each interaction that are shared between the Paf1C and SKI complex interfaces. Parenthetical letters on the diagram indicate the WDR61 chain examined. (D) AlphaFold2 structure of hWDR61 downloaded from the AlphaFold Protein Database with B-factors recoded to display the conservation scores of WDR61 calculated as in Figure 4 from RefSeq metazoan (left) or fungal (right) proteome databases.

This would be consistent with at least three non-exclusive possibilities. 1) The lack of WDR61 inclusion in Paf1C is a recent phenomenon in the evolutionary history of *S. cerevisiae* and most of the fungal WDR61 orthologs informing the conservation score still conserve these residues, 2) the residues that are truly instrumental to the WDR61-CTR9 interaction are primarily a subset of those involved with the WDR61-SKI complex interaction and thus would appear highly conserved even if CTR9 interaction was lost or 3) these residues are under other selective pressures, e.g. to maintain stability or localization of WDR61.

We additionally examined the potential for WDR61 inclusion in Paf1C by screening co-fold iPTMs between paired WDR61-Ctr9 orthologs across the Tree of Life. We hypothesized that if the WDR61-Ctr9 interaction was selectively lost in fungi or a subset of fungal species, we would expect to see a decreased iPTM score between fungal WDR61-Ctr9 homologs. We thus used our domain-centric HMM pipeline to detect WDR61 homologs by searching for the WD40 domain. Using this search, we detected 145 WDR61 homologs in the 304 species analyzed (**Figure R2A**, teal column). Importantly, while WDR61 homologs were detected in most fungi, our pipeline was unable to identify WDR61 homologs in 4 out of 6 Ascomycota species and several other clades. The variable detection of WDR61 in Ascomycota was unexpected, as we identified WDR61 homologs in ascomycete proteomes using BLAST (**Figure R1D**). We hypothesize that, given the generic nature of WD40 domains and the lack of another diagnostic domain for WDR61, our pipeline is not well-suited for the detection of WDR61 across a broad range of species.

As the reviewer points out, previous studies indicate that WDR61 is likely a component of Paf1C and the SKI complex in other clades, like plants (Oh *et al.*, 2004; Dorcey *et al.* 2012*)*. We thus wanted to test if the WDR61-Ctr9 interaction is predicted in organisms other than metazoans. Out of all the proteomes where WDR61 was detected, 133/145 have a putative Ctr9 ortholog (**Figure R2A,** orange column). We paired Ctr9 and WDR61 homologs from these species and performed an AlphaFold3 co-fold screen. Each protein pair was folded using 10 seeds (10, 15, 500, 43, 57, 5967, 4327, 798, 111, 287), resulting in 50 predicted models and iPTM scores for each protein pair (**Figure R2A,** heatmap). Consistent with previous work, we detected a high iPTM between *A. thaliana* homologs of WDR61 and Ctr9. The predicted model indicates that the mode of interaction between *A. thaliana* homologs is like that observed in cryo-EM structures of human WDR61-Ctr9 (**Figure R2B**). Additionally, we noticed that WDR61-Ctr9 homologs in non-Ascomycota fungal species, when co-folded, have a high iPTM (**Figure R2A**).

Based on these observations we conclude that we would be poorly positioned to propose a model describing the history of WDR61's inclusion in Paf1C that goes beyond what is currently available in the literature. However, we have amended the text to acknowledge that existing data suggest WDR61 inclusion in Paf1C predates the divergence of Opisthokonta and Viridiplantae clades and appears to have been lost in some fungi (pg. 6 lines 132-137 and pg.

24 lines 724-728). We stress that this hypothesis would have to be rigorously tested by carefully identifying WDR61 homologs in other fungal species followed by biochemical experiments.

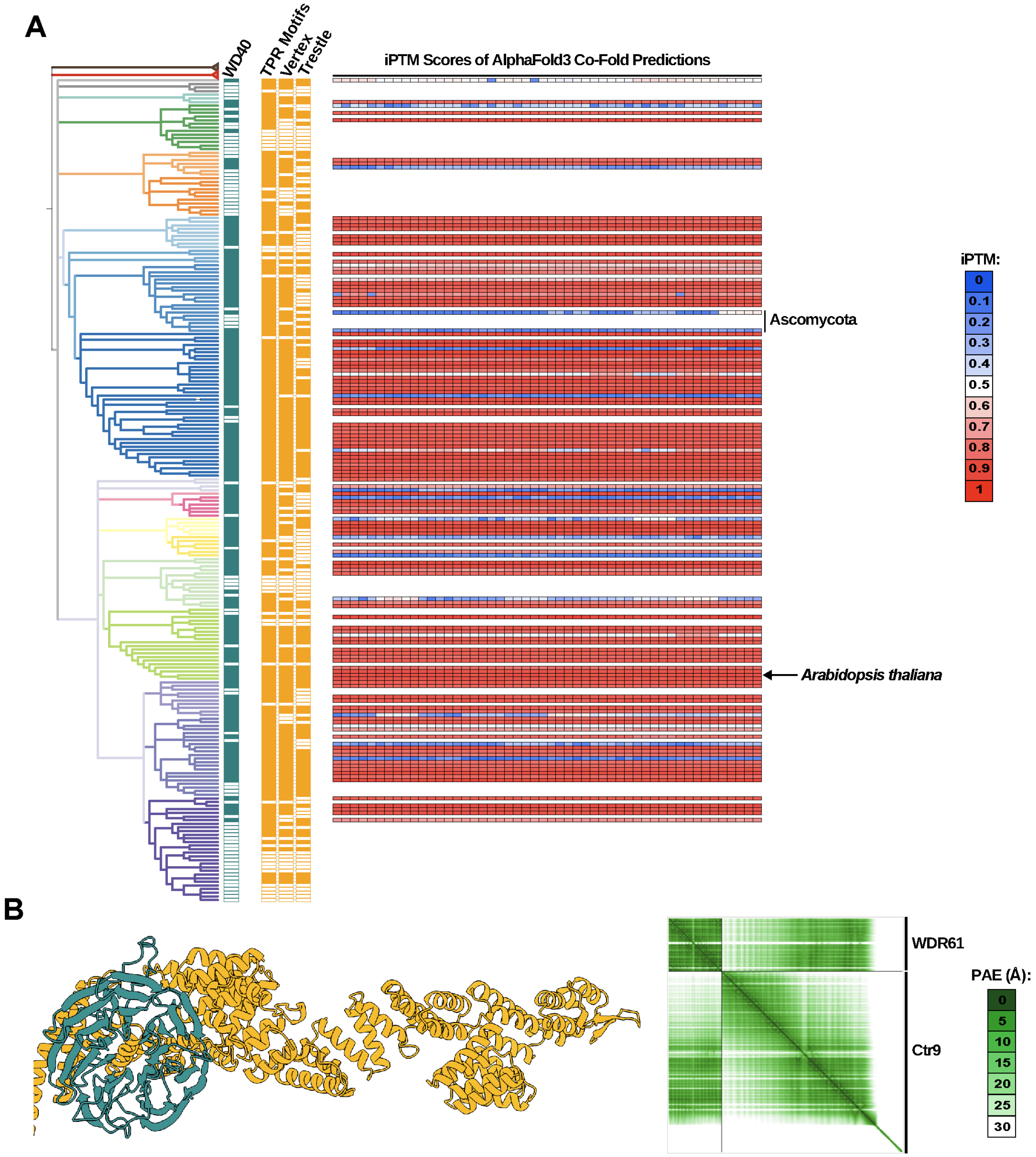

Figure R2. Detection of WDR61 homologs across 304 species. (A) Phylogenetic tree depicting the detection status of WDR61 and Ctr9 in the combined EukProt/GTDB database. Archaeal and Bacterial clades have been collapsed for visualization purposes, as no WDR61 homolog was detected in these clades. Each row corresponds to a proteome from an organism. A filled box indicates that the domain was detected in the proteome of the organism. Teal column indicates detection of the WDR61 WD40 domain and orange column indicates the detection of Ctr9 domains. Heatmap indicates iPTM values for 50 models generated by AlphaFold3. Each column in the heatmap corresponds to a single model. (B) AlphaFold3 predicted structure of *A. thaliana* WDR61-Ctr9. AtCtr9 is represented in orange and AtWDR61 is represented in teal. PAE plot of the model is shown on the right.

Dorcey, Eavan, et al. "Context-dependent dual role of SKI8 homologs in mRNA synthesis and turnover." PLoS genetics 8.4 (2012): e1002652.
Kögel, Alexander, et al. "The human SKI complex regulates channeling of ribosome-bound RNA to the exosome via an intrinsic gatekeeping mechanism." Molecular cell 82.4 (2022): 756-769.
Oh, Sookyung, et al. "A mechanism related to the yeast transcriptional regulator Paf1c is required for expression of the Arabidopsis FLC/MAF MADS box gene family." The Plant Cell 16.11(2004): 2940-2953.
Vos, Seychelle M., et al. "Structure of complete Pol II–DSIF–PAF–SPT6 transcription complex reveals RTF1 allosteric activation." Nature Structural & Molecular Biology 27.7 (2020): 668-677.
Zou, Xu-Dong, et al. "Genome-wide analysis of WD40 protein family in human." *Scientific reports* 6.1 (2016): 39262.

-Figure 5: My understanding of Figure 5 is that the authors are comparing relative rates of co-evolution between factors. I am surprised to see that several interactions are not found with their Z-score cutoff approach. This includes interactions between RNA polymerase II subunits RPB3-11, which are known to form a very stable heterodimer, and PAF1 and CTR9, which are known to interact quite robustly via the PAF1 N-terminus. Finally, ELF1 makes extensive contacts with RBP1, RPB2 and SPT5. It is thus surprising to not see any kind of co-evolution. Is this due to the Z-score cutoff or am I missing something more generally?

The reviewer is correct that some known physical interactions do not appear above the cutoff threshold for Evolutionary Rate Covariation (ERC). ERC is not a measure of amino acid co-evolution at interfaces. Our recent publication establishes that the correlation does not typically result from compensatory amino acid changes, as in co-evolution (Little, Chikina, and Clark. eLife 2024). Rather, ERC measures the correlation of the species-specific rates of evolution between a pair of proteins. This rate correlation often arises between co-functional proteins due to their shared selective pressures (Clark et al. Genome Research 2012; Clark et al. Genetics 2013; Little et al. Genome Research 2025).

The utility of ERC is to infer new functional interactions by identifying new hypotheses for co-functional proteins. It will sometimes not score known co-functional proteins highly because some proteins are influenced by other selective pressures, such as from additional roles in the cell. Like any screening technique, there are blind spots; however, high-scoring pairs are nevertheless valuable to formulate new hypotheses. ERC has thus been used to successfully identify new functional interactions in many contexts, from yeast to malaria to mammals (listed below). We have revised the manuscript to better reflect these goals and caveats (pg. 22 lines 662-666).

Clark, Nathan L., Eric Alani, and Charles F. Aquadro. "Evolutionary Rate Covariation in Meiotic Proteins Results from Fluctuating Evolutionary Pressure in Yeasts and Mammals." *Genetics* 193, no. 2 (2013): 529–38.
Clark, Nathan L., Eric Alani, and Charles F. Aquadro. "Evolutionary Rate Covariation Reveals Shared Functionality and Co-Expression of Genes." *Genome Research*, 2012.
Findlay, Geoffrey D., Jessica L. Sitnik, Wenke Wang, Charles F. Aquadro, Nathan L. Clark, and Mariana F. Wolfner. "Evolutionary Rate Covariation Identifies New Members of a Protein Network Required for Drosophila Melanogaster Female Post-Mating Responses." *PLOS Genetics* 10, no. 1 (2014): e1004108.
Hopson, Helena D., Radoslaw Igor Omelianczyk, Anayansi Ramirez, et al. "Evolutionary Rate Covariation across Malaria Parasite Species Enables Inference of Protein Interactions." *bioRxiv: The Preprint*

Server for Biology, January 30, 2026, 2026.01.29.702598. https://doi.org/10.64898/2026.01.29.702598.
Little, Jordan, Maria Chikina, and Nathan L. Clark. "Evolutionary Rate Covariation Is a Reliable Predictor of Co-Functional Interactions but Not Necessarily Physical Interactions." *Elife* 12 (2024): RP93333.
Little, Jordan H., Guillermo Hoffmann Meyer, Aakash Grover, et al. "ERC2.0 Evolutionary Rate Covariation Update Improves Inference of Functional Interactions across Large Phylogenies." *Genome Research*, ahead of print, August 7, 2025. https://doi.org/10.1101/gr.280586.125.
Raza, Qanber, Jae Young Choi, Yang Li, et al. "Evolutionary Rate Covariation Analysis of E-Cadherin Identifies Raskol as a Regulator of Cell Adhesion and Actin Dynamics in Drosophila." *PLoS Genetics* 15, no. 2 (2019): e1007720.
Thorpe, Holly J., Raghavendran Partha, Jordan Little, Nathan L. Clark, and Clement Y. Chow. "Evolutionary Rate Covariation Is Pervasive between Glycosylation Pathways and Points to Potential Disease Modifiers." *PLOS Genetics* 20, no. 9 (2024): e1011406. https://doi.org/10.1371/journal.pgen.1011406.

Minor comments

-Line 453- the authors mention that it is unclear whether the C-terminus of RTF1 interacts with CTR9 in humans. Doi: 10.1038/s41594-020-0437-1 found in cross linking mass spectrometry experiments that CTR9 and the RTF1 C-terminus were in proximity. The authors should consider rephrasing to indicate there is some data to support that the two could associate.

We thank the reviewer for bringing these observations to our attention. We have rephrased the text to acknowledge these data (pg. 21 lines 627-628), which we believe strengthen the argument for a conserved binding modality of the RTF1 Hook to Ctr9.

-Line 467-468: The authors state that the CTR9-RTF1 interactions may be weaker in humans. Is there any suggestion from the crystal structures or their evolutionary analysis why this might be the case? Which substitutions might drive this difference?

We thank the reviewer for the suggestions, but we prefer to be cautious in our interpretations. Ultimately, this is a question best addressed with biochemistry and since we have been unable to identify any obvious structural differences that could account for the discrepancy, we have tempered the language regarding this hypothesis.

-Figure 2: The authors should provide a key in the figure for panel A about what filled versus unfilled boxes mean. For panel B, AlphaFold structures would benefit from pLDDT coloring to indicate which regions are more reliable.

The wording in the Figure 2 legend has been changed to increase clarity regarding panel A. We have replaced Figure 2B with the pLDDT-colored structures to increase transparency regarding the confidence of the folded structures in these organisms.

REVIEWER #2

The manuscript titled "Evolutionary conservation and innovations of RNA polymerase II transcription elongation factors" presents a comprehensive and technically sophisticated evolutionary analysis of ten core RNA polymerase II transcription elongation factors (TEFs) across the Tree of Life. Using a domain-centric pipeline integrating structural prediction, and evolutionary rate covariation (ERC), the authors demonstrate that all ten TEFs were likely present in the last eukaryotic common ancestor, while also uncovering lineage-specific domain losses and innovations. The analyses of residue-level conservation and clade-specific divergence, particularly the comparison between fungal and metazoan homologs, generate testable hypotheses about metazoan-specific regulatory interfaces (e.g., in Cdc73, Ctr9, and Paf1). Overall, this manuscript is technically sound, of interest to the transcription field, and is a strong candidate for PLOS Biology. However, I do need the following comments addressed before accepting for publication.

1. The authors used a domain-centered approach to identify orthologs. How does the method differ from a Pfam scan? And what's the advantage over Pfam scan? Afterall, Pfam scan also uses HMM.

It is correct that PFAM scans use HMM builds informed by “signatures” of proteins with shared evolutionary origins. These tools are undoubtedly useful and effective. However, we know that PFAM can group functionally distinct, but frequently co-occurring, regions into a single HMM entity or omit known regions with known functions in their HMMs. For example, the PFAM Paf1 signature (PF03985) does not discriminate the functionally distinct Ctr9-binding, RNAPII-interacting, and Leo1-binding regions of the protein. Additionally, the only PFAM signature that is specific to Rtf1 recognizes the Plus-3 domain (PF03126) but omits regions like the HMD. Thus, any orthologous Rtf1 genes where the Plus-3 was exceedingly diverged or lost would be entirely undetectable when searching using the standard PFAM families. In analyzing the evolutionary history, conservation, and distribution of TEFs, we were aiming for an approach that required few assumptions. To reduce the potential for detection bias from rearranged, truncated, or otherwise divergent-but-homologous domain architectures, we conducted this study with a domain-first approach. We have added language to the manuscript to better communicate our rationale (pg. 7 lines 156-164).

2. Controversial to the authors' claim, not all the Prokaryotic proteomes selected for analysis are "NCBI type material", such as GB_GCA_001683985.1. And not all genomes are complete genomes. A high-quality complete genome is critical for the presence-absence analysis. If no complete genomes are available, the presence of known essential genes should be probed in the low-quality proteomes, which will increase the confidence in the conclusions of this manuscript.

We are grateful to the reviewer for noticing this discrepancy. While we curated reference prokaryotes from the GTDB web application by filtering for both “GTDB species representatives only” and “NCBI type material only”, we have re-examined these functions and notice that these settings correctly filter for GTDB species representatives, but not NCBI type material (tested on Google Chrome and Safari). We have corrected the methods to reflect the nature of the selection process.

The completeness of the proteomes used in this study is certainly a limitation and essential context for interpretation. To assess the completeness of the prokaryotic genomes used to

derive proteome data, we provide an analysis of the CheckM2 reference genome quality estimates provided by GTDB (Fig S4C). The introduction of CheckM scores established a "Controlled vocabulary of draft genome quality" wherein CheckM genome completeness estimates >70% are considered "Substantially Complete" and reflects a weighted assessment of lineage-specific sets of marker genes (Parks et al. 2025). The CheckM2 scores rely on a Machine Learning methodology that outperforms CheckM on prokaryotic genomes that may have undergone reductive evolution or have small genome sizes (Chklovski et al. 2023). Likewise, we provide two analyses of the potential for completeness of eukaryotic proteomes to lead to a bias in presence-absence determinations as evaluated by Benchmarking Universal Single-Copy Ortholog (BUSCO) scores (Fig S4B and S6B) (Simão et al. 2015). We believe these values - CheckM2 estimates and BUSCO scores - reflect the ability to detect known essential genes in the represented proteomes more accurately than can be practically examined in the scope of this study. We have amended text to refer to the threshold of CheckM2 completeness estimates >70% as "Substantially Complete" and to increase the clarity in the meaning of BUSCO scores by explicitly spelling out the acronym (pg. 45, lines 1427, 1432-1439).

Parks, Donovan H., et al. "CheckM: assessing the quality of microbial genomes recovered from isolates, single cells, and metagenomes." Genome research 25.7 (2015): 1043-1055.
Chklovski, Alex, et al. "CheckM2: a rapid, scalable and accurate tool for assessing microbial genome equality using machine learning." *Nature methods* 20.8 (2023): 1203-1212.
Simão, Felipe A., et al. "BUSCO: assessing genome assembly and annotation completeness with single-copy orthologs." Bioinformatics 31.19 (2015): 3210-3212.

3. Figure 1B shows the percentage of species that contain certain domains. One should be careful when making this kind of conclusion, especially in cases of small number of representatives are used, such as only 1 or 2 species are used in many clades. Similarly, the findings in Figure 2A need to be further confirmed by searching against more representative proteomes in Discoba and Metamonada.

The reviewer is correct to state that the small number of proteomes within some clades limits conclusions about putative domain loss events. For this reason, we performed expanded searches for potential orthologs with domains under-detected in metamonad, discobid, and alveolate proteomes using FoldSeek, AlphaFold and PSI-BLAST (S1 Table). We have also amended the text to be clearer about the limitations of the analysis and to be more explicit regarding the nature of these expanded searches (pg. 10 lines 290-293).

Based on the reviewer's suggestion, we also downloaded additional proteomes corresponding to Parabasilia and Fornicata sub-clades of Metamonada, Kinetoplastea and Diplonemea sub-clades of Discoba, and the Dinoflagellate sub-clade of Alevolata from NCBI. We then searched these proteomes using our domain-specific HMM approach. These searches were carried out for domains that were undetected in the above-mentioned sub-clades by any of the previously used approaches. Interestingly, while in most cases, we were still unable to detect certain domains, we were able to detect the Paf1 Leo1-binding domain, Ctr9 TPR motifs, and Leo1 Paf1-binding domain in the Fornicata sub-clade of Metamonads. Similarly, we were also able to detect the Rtf1 HMD in 1 dinoflagellate species. We have amended the text and S1 Table to include these new observations (pg. 11 lines 305-313, pg. 11 lines 319-324, and pg. 12 lines 387-388). We have further modified Fig 6 to reflect these new results.

4. More explanations about cutoff choices: 1) How are all the e-values decided? 2) Line 285:

"conservation score > 0.28", how is the 0.28 cutoff decided?

We chose the e-values based on prior studies using similar approaches (Culbertson and Levin, 2023; Lucas et al. 2023). We have added citations that reflect this to the main text (pg. 30 lines 863-865).

The conservation score cutoff of 0.28 was chosen because we deemed it to best reflect known molecular interfaces across all TEFs in the PDB:9EH2 elongation complex. However, it is certainly possible that some real, yet unknown interfaces could have a lower conservation score and thus remain under-emphasized by this specific approach. We have changed the text to better communicate our rationale (pg. 14 lines 443-444).

Culbertson, Edward M., and Tera C. Levin. "Eukaryotic CD-NTase, STING, and viperin proteins evolved via domain shuffling, horizontal transfer, and ancient inheritance from prokaryotes." PLoS biology 21.12 (2023): e3002436.
Lucas, Sarah AM, et al. "Highly dynamic gene family evolution suggests changing roles for PON genes within metazoa." Genome Biology and Evolution 15.2 (2023): evad011.

Minor comments:
1) Line 121: References for "Previous investigations" please. List one or two references.

References have been added to the main text as requested.

2) Figure 1A: Please consider coloring Paf1C in the same background color, so readers who are not familiar with Eukaryotic biology can catch the main point easily.

Since we have tried to keep colors consistent throughout the manuscript and have used many already, we opted for drawing a box around Paf1C and DSIF factors to emphasize their inclusion within complexes in Figure 1A. We hope this satisfactorily addresses the request.

3) Please comment on the observation that some bacteria lack the Spt5 homologs. Because NusG (Spt5) is thought to be present in all bacteria except endosymbionts (PMID: 33109766).

We thank you for raising this interesting point, which we now address in the Discussion of the revised manuscript (pg. 25 lines 742-744).

4) Figure 1B: How is the Tree of Life inferred? Or where does it come from?

The Tree of Life we display in this study is derived from the taxonomic classifications provided by GTDB and EukProt for prokaryotic and eukaryotic assemblies, respectively. We have clarified this in the Figure 1 legend.

5) Figure 3A: explain the conservation scores in the legend.

We have added text in the Figure 3A legend to spell out RER as “Relative Evolutionary Rate” and to more clearly communicate the nature of conservation scores.

6) Are the conservation scores in Figure 4 the same as the scores in Figure 3?

No, the conservation scores in Figure 4 reflect those calculated amongst metazoan orthologs of human TEF sequences or fungal orthologs of *S. cerevisiae* TEF sequences. We have changed the text to be more explicit about this.

7) Figure S4E shows ~20% Asgardarchaeota proteomes contain Spt4, but the percentage seems to be higher than 20% in Figure S5.

Good catch! The lower detection statistics are a reflection of the different search approach we used to look for orthologs in Asgardarcheota, where we searched the proteomes using each domain-specific HMM and performed a cursory validation via reciprocal blast. In the previous Figure S4E, we had not re-examined “Potential” and “Dubious” hits that had not identified the known orthologs in the reciprocal BLAST, as we had done for our other searches. After performing a re-analysis consistent with the methodology employed throughout the manuscript, we detect orthologs of Spt5 (174/218 proteomes), Spt4 (157/218 proteomes), and Elf1 (143/218 proteomes) in a greater number of proteomes. All other TEF domains remained undetected even after re-visiting the “Potential” and “Dubious” hits. We updated the Figure S4E accordingly.

8) For all Alphafold predictions, pLDDT or other confidence metrics should be included next to the predicted structures.

In Figure 2B, the structures have now been colored with the pLDDT score. For AlphaFold predicted structures displayed in Fig 4 and S9 Fig, since we are limited in space, we have displayed the AlphaFold structures colored by pLDDT scores separately in a new supplementary figure (**Fig R3**). This figure has been added as Figure S10 in the manuscript, and the text has been modified accordingly.

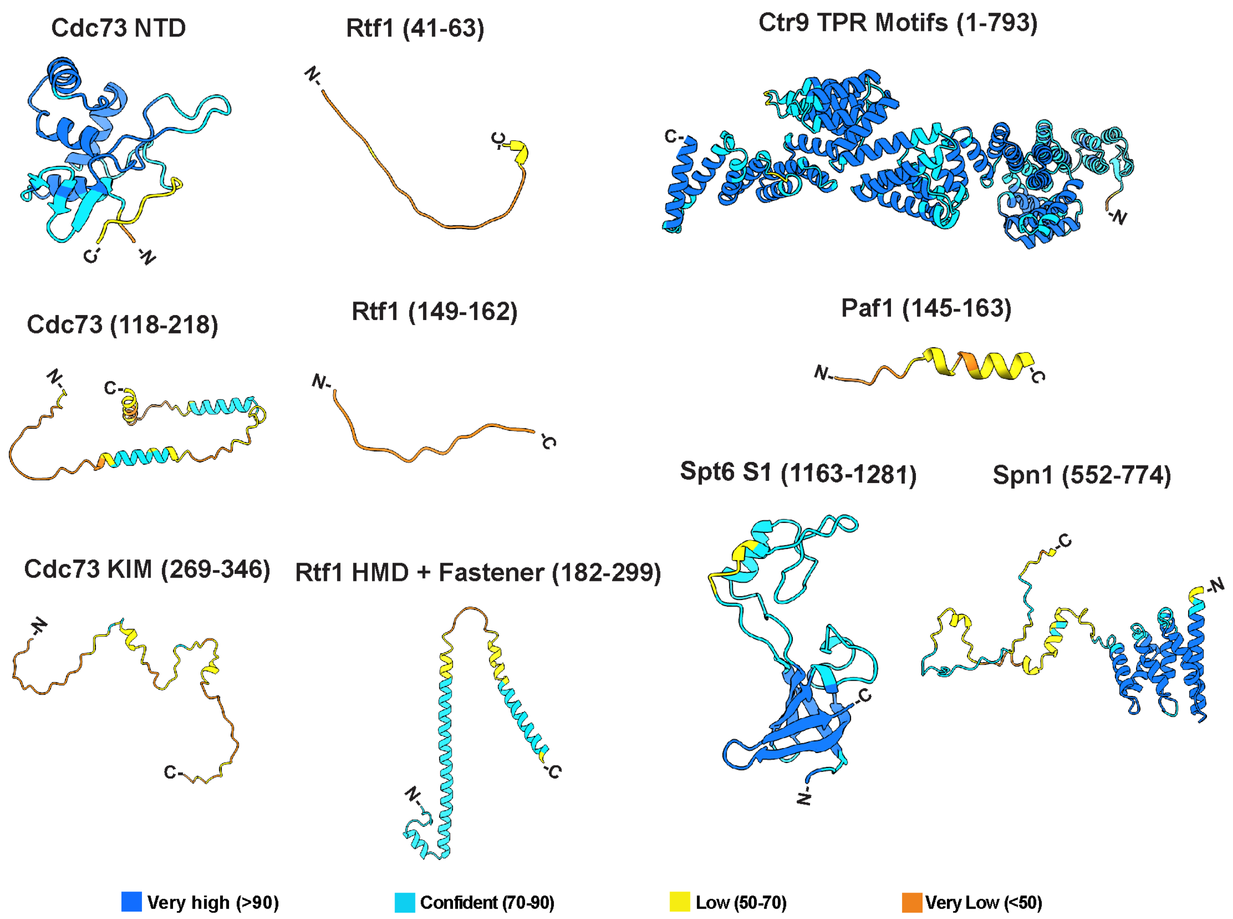

R3 Fig (same as S10 Fig). TEF AlphaFold3 Monomer Predictions Colored by pLDDT. AlphaFold2 predicted structures of H. sapiens TEFs indicated in Fig 4 and Fig S9 colored by pLDDT scores of residues.

9) Only AlphaFold3 is described in the Method, but in the maintext, usage of AlphaFold2 is mentioned at several places. Please confirm this is correct and add AlphaFold2 to Method.

Monomeric TEF structures represented in Figure 4 and Figure S9 were downloaded from the AlphaFold Protein Structure Database, which were computed with AlphaFold2. All novel structural predictions we make in this study were generated using AlphaFold3. We have changed the data retrieval section of the Methods to explicitly state that protein structures downloaded from the AlphaFold database were pre-computed using AlphaFold2.

10) Are the predicted structures relaxed? This information needs to be included in the Method.

The AlphaFold2 predictions were downloaded from the AlphaFold Protein Structure Database which had previously subjected them to energy minimization. All novel AlphaFold3 structures presented in this manuscript were not relaxed. We have included this information in the methods (pg. 34 lines 963-964).

11) The "eukprotAnnotations.csv" also contains Archaea and Bacteria.

We thank the reviewer for noticing this. The file has been renamed to reflect the inclusion of GTDB species annotations used in the broad Tree of Life searches.

12) Interpretation of domain "loss" versus extreme divergence. While the authors appropriately acknowledge technical limitations, several conclusions (e.g., loss of Rtf1 HMD or Cdc73 NTD in Kinetoplastea/Diplonemea) are framed somewhat strongly. It would help to: More consistently distinguish between bona fide loss and divergence beyond detection. Explicitly label these cases as "putative losses" throughout the Results and Discussion.

We thank the reviewer for recognizing our efforts to acknowledge the technical limitations of our approaches. To ensure clarity and accuracy in our conclusions, we have attempted to temper the language particularly surrounding interpretation of the putative loss of Rtf1 HMD and Cdc73 NTD in Kinetoplastea and Diplonemea clades (pg. 10 lines 290-293).
