# Supplementary material for: Evolutionary analysis of transcription elongation factors reveals conserved and lineage-specific regulatory domains
Source: PLoS Biol. 2026 Jun 15;24(6):e3003855. doi: 10.1371/journal.pbio.3003855 (PMC13289920; doi:10.1371/journal.pbio.3003855)
Supplement: Peer review history

# Editor report: Decision Letter 0

**DOI:** 10.1371/journal.pbio.3003855.r001

**Authors:** Richard Hodge

9 Jan 2026

Dear Dr Arndt,

Thank you for submitting your manuscript entitled "Evolutionary conservation and innovations of RNA polymerase II transcription elongation factors" for consideration as a Research Article by PLOS Biology. Please accept my sincere apologies for the delay in getting back to you due to the closure of the editorial office during the recent holiday period.

Your manuscript has now been evaluated by the PLOS Biology editorial staff and I am writing to let you know that we would like to send your submission out for external peer review.

IMPORTANT - Please note that we have not yet obtained advice from an academic editor with relevant expertise about your submission. Therefore, we have not been able to take a firm editorial stance about the degree of advance offered by the findings and we will be looking for some enthusiasm from the reviewers during the peer review process.

Before we can send your manuscript to reviewers, we need you to complete your submission by providing the metadata that is required for full assessment. To this end, please login to Editorial Manager where you will find the paper in the 'Submissions Needing Revisions' folder on your homepage. Please click 'Revise Submission' from the Action Links and complete all additional questions in the submission questionnaire.

Once your full submission is complete, your paper will undergo a series of checks in preparation for peer review. After your manuscript has passed the checks it will be sent out for review. To provide the metadata for your submission, please Login to Editorial Manager (https://www.editorialmanager.com/pbiology) within two working days, i.e. by Jan 11 2026 11:59PM.

If your manuscript has been previously peer-reviewed at another journal, PLOS Biology is willing to work with those reviews in order to avoid re-starting the process. Submission of the previous reviews is entirely optional and our ability to use them effectively will depend on the willingness of the previous journal to confirm the content of the reports and share the reviewer identities. Please note that we reserve the right to invite additional reviewers if we consider that additional/independent reviewers are needed, although we aim to avoid this as far as possible. In our experience, working with previous reviews does save time.

If you would like us to consider previous reviewer reports, please edit your cover letter to let us know and include the name of the journal where the work was previously considered and the manuscript ID it was given. In addition, please upload a response to the reviews as a 'Prior Peer Review' file type, which should include the reports in full and a point-by-point reply detailing how you have or plan to address the reviewers' concerns.

During the process of completing your manuscript submission, you will be invited to opt-in to posting your pre-review manuscript as a bioRxiv preprint. Visit http://journals.plos.org/plosbiology/s/preprints for full details. If you consent to posting your current manuscript as a preprint, please upload a single Preprint PDF.

Feel free to email us at plosbiology@plos.org if you have any queries relating to your submission.

Kind regards,

Richard

Richard Hodge, PhD

Senior Editor, PLOS Biology

rhodge@plos.org

PLOS

Empowering researchers to transform science

Carlyle House, Carlyle Road, Cambridge, CB4 3DN, United Kingdom

California (U.S.) corporation #C2354500, based in San Francisco

# Peer review: Decision Letter 1

**DOI:** 10.1371/journal.pbio.3003855.r002

**Authors:** Richard Hodge

20 Feb 2026

Dear Dr Arndt,

Thank you for your patience while your manuscript "Evolutionary conservation and innovations of RNA polymerase II transcription elongation factors" went through peer-review at PLOS Biology. Please accept my sincere apologies for the delays that you have experienced during the peer review process. Your manuscript has now been evaluated by the PLOS Biology editors, an Academic Editor with relevant expertise, and by two independent reviewers. Please note that we had recruited a third reviewer to provide a review of your manuscript, but they are now late in submitting their comments so we are moving forward to a decision to avoid further loss of time.

As you will see in the reviewer reports at the end of the this e-mail, both reviewers are supportive of the manuscript and think it will be a valuable resource to the field. Therefore, we are pleased to offer you the opportunity to address the comments from the reviewers in a revision that we anticipate should not take you very long. We will then assess your revised manuscript and your response to the reviewers' comments with our Academic Editor aiming to avoid further rounds of peer-review, although we might need to consult with the reviewers, depending on the nature of the revisions.

In addition to these revisions, you may need to complete some formatting changes, which you will receive in a follow up email. A member of our team will be in touch with a set of requests shortly. If you do not receive a separate email within a few days, please assume that checks have been completed, and no additional changes are required.

We expect to receive your revised manuscript within 2 months. Please email us (plosbiology@plos.org) if you have any questions or concerns, or would like to request an extension.

At this stage, your manuscript remains formally under active consideration at our journal; please notify us by email if you do not intend to submit a revision so that we withdraw the manuscript.

**IMPORTANT - SUBMITTING YOUR REVISION**

Your revisions should address the specific points made by each reviewer. Please submit the following files along with your revised manuscript:

1. A 'Response to Reviewers' file - this should detail your responses to the editorial requests, present a point-by-point response to all of the reviewers' comments, and indicate the changes made to the manuscript.

*NOTE: In your point-by-point response to the reviewers, please provide the full context of each review. Do not selectively quote paragraphs or sentences to reply to. The entire set of reviewer comments should be present in full and each specific point should be responded to individually.

You should also cite any additional relevant literature that has been published since the original submission and mention any additional citations in your response.

2. In addition to a clean copy of the manuscript, please also upload a 'track-changes' version of your manuscript that specifies the edits made. This should be uploaded as a "Revised Article with Changes Highlighted " file type.

*Resubmission Checklist*

When you are ready to resubmit your revised manuscript, please refer to this resubmission checklist: https://plos.io/Biology_Checklist

To submit a revised version of your manuscript, please go to https://www.editorialmanager.com/pbiology/ and log in as an Author. Click the link labelled 'Submissions Needing Revision' where you will find your submission record.

Please make sure to read the following important policies and guidelines while preparing your revision:

*Published Peer Review*

Please note while forming your response, if your article is accepted, you may have the opportunity to make the peer review history publicly available. The record will include editor decision letters (with reviews) and your responses to reviewer comments. If eligible, we will contact you to opt in or out. Please see here for more details:

https://blogs.plos.org/plos/2019/05/plos-journals-now-open-for-published-peer-review/

*PLOS Data Policy*

Please note that as a condition of publication PLOS' data policy (http://journals.plos.org/plosbiology/s/data-availability) requires that you make available all data used to draw the conclusions arrived at in your manuscript. If you have not already done so, you must include any data used in your manuscript either in appropriate repositories, within the body of the manuscript, or as supporting information (N.B. this includes any numerical values that were used to generate graphs, histograms etc.). For an example see here: http://www.plosbiology.org/article/info%3Adoi%2F10.1371%2Fjournal.pbio.1001908#s5

*Blot and Gel Data Policy*

We require the original, uncropped and minimally adjusted images supporting all blot and gel results reported in an article's figures or Supporting Information files. We will require these files before a manuscript can be accepted so please prepare them now, if you have not already uploaded them. Please carefully read our guidelines for how to prepare and upload this data: https://journals.plos.org/plosbiology/s/figures#loc-blot-and-gel-reporting-requirements

*Protocols deposition*

To enhance the reproducibility of your results, we recommend that if applicable you deposit your laboratory protocols in protocols.io, where a protocol can be assigned its own identifier (DOI) such that it can be cited independently in the future. Additionally, PLOS ONE offers an option for publishing peer-reviewed Lab Protocol articles, which describe protocols hosted on protocols.io. Read more information on sharing protocols at https://plos.org/protocols?utm_medium=editorial-email&utm_source=authorletters&utm_campaign=protocols

Thank you again for your submission to our journal. We hope that our editorial process has been constructive thus far, and we welcome your feedback at any time. Please don't hesitate to contact us if you have any questions or comments.

Best wishes,

Richard

Richard Hodge, PhD

Senior Editor, PLOS Biology

rhodge@plos.org

----------------------------------------------------------------

REVIEWS:

Reviewer #1: This manuscript investigates the evolutionary relationships between transcription elongation factors using domain centric method to identify homologs. The authors clearly explain their rational and approach, and make interesting observations about relative conservation and origin of transcription elongation factors. The manuscript makes some compelling observations, including a putative zinc binding domain on SPT6. Overall, the manuscript will be a useful resource for anyone studying transcription elongation and provides new avenues for research. I have a few comments the authors should address prior to publication.

-The authors omitted WDR61 from their analysis. WDR61 is not a component of yeast PAF1c, but there is a possibility that it is a member of PAF1c in other organisms outside of human. The way that WDR61 interacts with human CTR9 would preclude it from being in the SKI RNA surveillance complex. This suggests that WDR61 is able to participate in multiple complexes that are perhaps mutually exclusive. It thus would be nice if the authors could use their approach to indicate whether WDR61 is a constitutive member of PAF1c or whether it only appears in select clades. It is understood that this analysis may be challenging.

-Figure 5: My understanding of Figure 5 is that the authors are comparing relative rates of co-evolution between factors. I am surprised to see that several interactions are not found with their Z-score cutoff approach. This includes interactions between RNA polymerase II subunits RPB3-11, which are known to form a very stable heterodimer, and PAF1 and CTR9, which are known to interact quite robustly via the PAF1 N-terminus. Finally, ELF1 makes extensive contacts with RBP1, RPB2 and SPT5. It is thus surprising to not see any kind of co-evolution. Is this due to the Z-score cutoff or am I missing something more generally?

Minor comments

-Line 453- the authors mention that it is unclear whether the C-terminus of RTF1 interacts with CTR9 in humans. Doi: 10.1038/s41594-020-0437-1 found in cross linking mass spectrometry experiments that CTR9 and the RTF1 C-terminus were in proximity. The authors should consider rephrasing to indicate there is some data to support that the two could associate.

-Line 467-468: The authors state that the CTR9-RTF1 interactions may be weaker in humans. Is there any suggestion from the crystal structures or their evolutionary analysis why this might be the case? Which substitutions might drive this difference?

-Figure 2: The authors should provide a key in the figure for panel A about what filled versus unfilled boxes mean. For panel B, AlphaFold structures would benefit from pLDDT coloring to indicate which regions are more reliable.

Reviewer #2: The manuscript titled "Evolutionary conservation and innovations of RNA polymerase II transcription elongation factors" presents a comprehensive and technically sophisticated evolutionary analysis of ten core RNA polymerase II transcription elongation factors (TEFs) across the Tree of Life. Using a domain-centric pipeline integrating structural prediction, and evolutionary rate covariation (ERC), the authors demonstrate that all ten TEFs were likely present in the last eukaryotic common ancestor, while also uncovering lineage-specific domain losses and innovations. The analyses of residue-level conservation and clade-specific divergence, particularly the comparison between fungal and metazoan homologs, generate testable hypotheses about metazoan-specific regulatory interfaces (e.g., in Cdc73, Ctr9, and Paf1). Overall, this manuscript is technically sound, of interest to the transcription field, and is a strong candidate for PLOS Biology. However, I do need the following comments addressed before accepting for publication.

1. The authors used a domain-centered approach to identify orthologs. How does the method differ from a Pfam scan? And what's the advantage over Pfam scan? Afterall, Pfam scan also uses HMM.

2. Controversial to the authors' claim, not all the Prokaryotic proteomes selected for analysis are "NCBI type material", such as GB_GCA_001683985.1. And not all genomes are complete genomes. A high-quality complete genome is critical for the presence-absence analysis. If no complete genomes are available, the presence of known essential genes should be probed in the low-quality proteomes, which will increase the confidence in the conclusions of this manuscript.

3. Figure 1B shows the percentage of species that contain certain domains. One should be careful when making this kind of conclusion, especially in cases of small number of representatives are used, such as only 1 or 2 species are used in many clades. Similarly, the findings in Figure 2A need to be further confirmed by searching against more representative proteomes in Discoba and Metamonada.

4. More explanations about cutoff choices: 1) How are all the e-values decided? 2) Line 285: "conservation score > 0.28", how is the 0.28 cutoff decided?

Minor comments:

1) Line 121: References for "Previous investigations" please. List one or two references.

2) Figure 1A: Please consider coloring Paf1C in the same background color, so readers who are not familiar with Eukaryotic biology can catch the main point easily.

3) Please comment on the observation that some bacteria lack the Spt5 homologs. Because NusG (Spt5) is thought to be present in all bacteria except endosymbionts (PMID: 33109766).

4) Figure 1B: How is the Tree of Life inferred? Or where does it come from?

5) Figure 3A: explain the conservation scores in the legend.

6) Are the conservation scores in Figure 4 the same as the scores in Figure 3?

7) Figure S4E shows ~20% Asgardarchaeota proteomes contain Spt4, but the percentage seems to be higher than 20% in Figure S5.

8) For all Alphafold predictions, pLDDT or other confidence metrics should be included next to the predicted structures.

9) Only AlphaFold3 is described in the Method, but in the maintext, usage of AlphaFold2 is mentioned at several places. Please confirm this is correct and add AlphaFold2 to Method.

10) Are the predicted structures relaxed? This information needs to be included in the Method.

11) The "eukprotAnnotations.csv" also contains Archaea and Bacteria.

12) Interpretation of domain "loss" versus extreme divergence. While the authors appropriately acknowledge technical limitations, several conclusions (e.g., loss of Rtf1 HMD or Cdc73 NTD in Kinetoplastea/Diplonemea) are framed somewhat strongly. It would help to: More consistently distinguish between bona fide loss and divergence beyond detection. Explicitly label these cases as "putative losses" throughout the Results and Discussion.

# Author comment: Author response to Decision Letter 2

**DOI:** 10.1371/journal.pbio.3003855.r003

30 Apr 2026

Attachment Submitted filename: Francette_Grover_ResponseToReviewers.pdf

# Peer review: Decision Letter 2

**DOI:** 10.1371/journal.pbio.3003855.r004

**Authors:** Richard Hodge

21 May 2026

Dear Dr Arndt,

Thank you for your patience while we considered your revised manuscript "Evolutionary conservation and innovations of RNA polymerase II transcription elongation factors" for publication as a Research Article at PLOS Biology. This revised version of your manuscript has been evaluated by the PLOS Biology editors, the Academic Editor and the original reviewers.

Based on the reviews, I am pleased to say that we are likely to accept this manuscript for publication, provided you satisfactorily address the following data and other policy-related requests that I have provided below (A-D):

(A) We would like to suggest a minor edit to the title, as follows, just to provide some more detail. I appreciate that ‘RNA Polymerase II’ has been removed from this version just to streamline it, but if you would prefer to keep then please feel free to add it back in. Please ensure you change both the manuscript file and the online submission system, as they need to match for final acceptance:

“Evolutionary analysis of transcription elongation factors reveals conserved and lineage-specific regulatory domains”

(B) Thank you for already providing the underlying data for the figures in the Zenodo repository (10.5281/zenodo.17856448). This looks good, but I would be grateful if it could be made clearer which data files relate to each specific figure so it is easier for readers to find the data. We require the individual numerical values that underlie the summary data displayed in the following figure panels:

Figure 1B, 2A, 2E, 3A, 3C, 4A, S2A-E, S3A-E, S4A-E, S5, S6A-B, S7A-B, S8A-C, S9A, S11C-D, S12C, S12E, S13A-B

(C) In addition, we note that the files in the Zenodo repository are currently restricted. I would be grateful if the data could be made publicly available at this stage.

(D) Please also ensure that each of the relevant figure legends in your manuscript include information on *WHERE THE UNDERLYING DATA CAN BE FOUND*, and ensure your supplemental data file/s has a legend.

------------------------------------------------------------------------

As you address these items, please take this last chance to review your reference list to ensure that it is complete and correct. If you have cited papers that have been retracted, please include the rationale for doing so in the manuscript text, or remove these references and replace them with relevant current references. Any changes to the reference list should be mentioned in the cover letter that accompanies your revised manuscript.

In addition to these revisions, you may need to complete some formatting changes, which you will receive in a follow up email. A member of our team will be in touch with a set of requests shortly. If you do not receive a separate email within a few days, please assume that checks have been completed, and no additional changes are required.

We expect to receive your revised manuscript within two weeks.

To submit your revision, please go to https://www.editorialmanager.com/pbiology/ and log in as an Author. Click the link labelled 'Submissions Needing Revision' to find your submission record. Your revised submission must include the following:

- a cover letter that should detail your responses to any editorial requests, if applicable, and whether changes have been made to the reference list

- a Response to Reviewers file that provides a detailed response to the reviewers' comments (if applicable, if not applicable please do not delete your existing 'Response to Reviewers' file.)

- a track-changes file indicating any changes that you have made to the manuscript.

NOTE: If Supporting Information files are included with your article, note that these are not copyedited and will be published as they are submitted. Please ensure that these files are legible and of high quality (at least 300 dpi) in an easily accessible file format. For this reason, please be aware that any references listed in an SI file will not be indexed. For more information, see our Supporting Information guidelines:

https://journals.plos.org/plosbiology/s/supporting-information

*Published Peer Review History*

Please note that you may have the opportunity to make the peer review history publicly available. The record will include editor decision letters (with reviews) and your responses to reviewer comments. If eligible, we will contact you to opt in or out. Please see here for more details:

https://plos.org/published-peer-review-history/

*Press*

Should you, your institution's press office or the journal office choose to press release your paper, please ensure you have opted out of Early Article Posting on the submission form. We ask that you notify us as soon as possible if you or your institution is planning to press release the article.

*Protocols deposition*

To enhance the reproducibility of your results, we recommend that if applicable you deposit your laboratory protocols in protocols.io, where a protocol can be assigned its own identifier (DOI) such that it can be cited independently in the future. Additionally, PLOS ONE offers an option for publishing peer-reviewed Lab Protocol articles, which describe protocols hosted on protocols.io. Read more information on sharing protocols at https://plos.org/protocols?utm_medium=editorial-email&utm_source=authorletters&utm_campaign=protocols

Please do not hesitate to contact me should you have any questions.

Best regards,

Richard

Richard Hodge, PhD

Senior Editor, PLOS Biology

rhodge@plos.org

------------------------------------------------------------------------

Reviewer remarks:

Reviewer #1: The authors have addressed all of my concerns.

Reviewer #2: No comments provided.

# Editor report: Decision Letter 3

**DOI:** 10.1371/journal.pbio.3003855.r005

**Authors:** Richard Hodge

31 May 2026

Dear Dr Arndt,

On behalf of my colleagues and the Academic Editor, Tom Misteli, I am pleased to say that we can accept your manuscript for publication, provided you address any remaining formatting and reporting issues. These will be detailed in an email you should receive within 2-3 business days from our colleagues in the journal operations team; no action is required from you until then. Please note that we will not be able to formally accept your manuscript and schedule it for publication until you have completed any requested changes.

Please take a minute to log into Editorial Manager at http://www.editorialmanager.com/pbiology/, click the "Update My Information" link at the top of the page, and update your user information to ensure an efficient production process.

PRESS

We frequently collaborate with press offices. If your institution or institutions have a press office, please notify them about your upcoming paper at this point, to enable them to help maximise its impact. If the press office is planning to promote your findings, we would be grateful if they could coordinate with biologypress@plos.org. If you have previously opted in to the early version process, we ask that you notify us immediately of any press plans so that we may opt out on your behalf.

We also ask that you take this opportunity to read our Embargo Policy regarding the discussion, promotion and media coverage of work that is yet to be published by PLOS. As your manuscript is not yet published, it is bound by the conditions of our Embargo Policy. Please be aware that this policy is in place both to ensure that any press coverage of your article is fully substantiated and to provide a direct link between such coverage and the published work. For full details of our Embargo Policy, please visit http://www.plos.org/about/media-inquiries/embargo-policy/.

Thank you again for choosing PLOS Biology for publication and supporting Open Access publishing. We look forward to publishing your study.

Best wishes,

Richard

Richard Hodge, PhD

Senior Editor, PLOS Biology

rhodge@plos.org

PLOS

Empowering researchers to transform science

Carlyle House, Carlyle Road, Cambridge, CB4 3DN, United Kingdom

California (U.S.) corporation #C2354500, based in San Francisco
